# Supplementary material for: Canonical and noncanonical Hippo signaling in C. elegans
Source: Genetics. 2026 Feb 26;233(1):iyag056. doi: 10.1093/genetics/iyag056 (PMC13147543; doi:10.1093/genetics/iyag056)
Supplement: iyag056_Supplementary_Data [file iyag056_supplementary_data.zip › 260310 composite Supp 4.pdf]

a

Lt blue: Ste20 S/T kinase
Yellow: SARAH coiled coil

CLUSTAL O(1.2.4) multiple sequence alignment

```
CeCST-1         --MPPST-----DSSRRNSEEGSSDGFKLDSSALNKPPEEVFDIVGKLGEGSYGSVHKAI     53
DmHpo           MSEPEVTSVVDMKSPNISSSCSFFKLKKLSEESLLQPPEKVFDIMYKLGEGSYGSVYKAV     60
HsMST1/STK3     -----------ME----QPPAPKSKLKKLSEDSLTKQPEEVFDVLEKLGEGSYGSVFKAI     45
HsMst2/STK4     --------METVQ----LRNPPRRQLKKLDEDSLTKQPEEVFDVLEKLGEGSYGSVYKAI     48
                            .           .  **...:* : **:***:: **********.**:

CeCST-1         HRESGHVLAIKKVPVDTDLQEIIKEISIMQQCKSKYVVKYYGSYFKHSDLWIVMEYCGAG     113
DmHpo           HKESSSIVAIKLVPVESDLHEIIKEISIMQQCDSPYVVRYYGSYFKQYDLWICMEYCGAG     120
HsMST1/STK3     HKESGQVVAIKQVPVESDLQEIIKEISIMQQCDSPYVVKYYGSYFKNTDLWIVMEYCGAG     105
HsMst2/STK4     HKETGQIVAIKQVPVESDLQEIIKEISIMQQCDSPHVVKYYGSYFKNTDLWIVMEYCGAG     108
                *:*:. ::*** ***::**:************.* :**:*******: **** *******

CeCST-1         SISDIMRARRKPLSEQEISAVLRDTLKGLQYLHDLKKIHRDIKAGNILLNTDGIAKLADF     173
DmHpo           SVSDIMRLRKKTLTEDEIATILSDTLQGLVYLHLRRKIHRDIKAANILLNTEGYAKLADF     180
HsMST1/STK3     SVSDIIRLRNKTLIEDEIATILKSTLKGLEYLHFMRKIHRDIKAGNILLNTEGHAKLADF     165
HsMst2/STK4     SVSDIIRLRNKTLTEDEIATILQSTLKGLEYLHFMRKIHRDIKAGNILLNTEGHAKLADF     168
                *:***:* *.* * *:**:::* .**:** ***   :*******.******:* ******

CeCST-1         GVAGQLTDTMAKRNTVIGTPFWMAPEVIEEIGYDTKADIWSLGITAIEMAEGRPPYSDIH     233
DmHpo           GVAGQLTDTMAKRNTVIGTPFWMAPEVIEEIGYDCVADIWSLGITALEMAEGKPPYGEIH     240
HsMST1/STK3     GVAGQLTDTMAKRNTVIGTPFWMAPEVIQEIGYNCVADIWSLGITSIEMAEGKPPYADIH     225
HsMst2/STK4     GVAGQLTDTMAKRNTVIGTPFWMAPEVIQEIGYNCVADIWSLGITAIEMAEGKPPYADIH     228
                ****************************:****:  *********::*****:***.:**

CeCST-1         PMRAIFMIPTKPPPTFKKPEEWSSEFNDFIRSCLIKKPEERKTALRLCEHTFIKNAPGCD     293
DmHpo           PMRAIFMIPQKPPPSFREPDRWSTEFIDFVSKCLVKEPDDRATATELLEHEFIRNAKHRS     300
HsMST1/STK3     PMRAIFMIPTNPPPTFRKPELWSDDFTDFVKKCLVKNPEQRATATQLLQHPFIKNAKPVS     285
HsMst2/STK4     PMRAIFMIPTNPPPTFRKPELWSDNFTDFVKQCLVKSPEQRATATQLLQHPFVRSAKGVS     288
                ********* :***:*::*: ** :* **: .**:*.*::* ** .* :* *::.*   .

CeCST-1         IMQLMIQDAQEKAILGQAPMA------AS-----------SGNDATLL----SEGMSTMI     332
DmHpo           ILKPMLEETCAIREQQRANRSFGGVLAASQAKSLAT--QENGMQQHITDNAFMEDPGTLV     358
HsMST1/STK3     ILRDLITEAMEIKAKRHEEQQ----------RELEE-EEENSDEDEL-------DSHTMV     327
HsMst2/STK4     ILRDLINEAMDVKLKRQESQQ----------REVDQDDEENSEEDEM-------DSGTMV     331
                *:: :: ::       :                       .. :  :       .  *::

CeCST-1         D--GG--------ESTLVQHKDNYVTA-----QSLR-----------------SQME---     357
DmHpo           PEKFGEYQQSSASDATMIAHAEQGVDEGTLGPGGLRNLSKAAAPAAASSAASPLDMPAVD     418
HsMST1/STK3     KTSV--------------------ESV-----GTMRATSTM------SEGA--QTMI---     351
HsMst2/STK4     RAVG--------------------DEM-----GTVRVASTM------TDGA--NTMI---     355
                                                  :*                   *
```

```
CeCST-1           -------SLRIGGEIPKS-AYSSSKNGNSPRVQPPGHTASASDPSKNQPFAQ----DGTG   405
DmHpo             SGTMVELESNLGSTMVINSDSDDSTTAKNNDDQKPR---NRYRPQFLEHFDRKNAGDGRG   475
HsMST1/STK3       EHNSTMLESDLG-TMVINSEDEEEEDGTMKRNATSP---QVQRPSFMDYFDKQDFKNKSH   407
HsMst2/STK4       EHDDT-LPSQLG-TMVINAEDEE-EEGTMKRRDETM---QPAKPSFLEYFEQKEKENQIN   409
                            :*  :  .   ..   ..           .   *.  : * :    :

CeCST-1           PNF------------------------------QLGTSE-----S----SY--------   417
DmHpo             DEKPIATEYSPAAAEQQQQQQQQQQQQQQDEQHLASGANDLNNWEHNMEMQFQQISAINQ   535
HsMST1/STK3       E----------------------------------------NCNQNM------------   414
HsMst2/STK4       S-----------------------------------------FGKSV------------   415


CeCST-1           -----------KDASYNMMNTE--------------------------------A----E   430
DmHpo             YGLQQHQQQQQVLMAYPLMNEQLIALNNQPNLLLSNAAPMGQQGIPAAAPAQPPPAYQNQ   595
HsMST1/STK3       ------------HEPF----------------------PMS-----------------KN   423
HsMst2/STK4       ------------PGP---------------------------------------------L   419


CeCST-1           YENRFQRAVVDGDFEFLRNITLDELIRRKESLDSEMEEEIRELQRRYKTKRQPILDVIEI   490
DmHpo             HMHTQSHAYVEGEFEFLKFLTFDDLNQRLCNIDHEMELEIEQLNKKYNAKRQPIVDAMNA   655
HsMST1/STK3       VFPDNWKVPQDGDFDFLKNLSLEELQMRLKALDPMMEREIEELRQRYTAKRQPILDAMDA   483
HsMst2/STK4       KNSSDWKIPQDGDYEFLKSWTVEDLQKRLLALDPMMEQEIEEIRQKYQSKRQPILDAIEA   479
                        :   :*:::**:  :.::*  *   :*  ** **.::.::* :*****:*.::

CeCST-1           KKRLQ----------   495
DmHpo             KRKRQQNINNNLIKI   670
HsMST1/STK3       KKRRQQN--------   490
HsMst2/STK4       KKRRQQNF-------   487
                  *:: *
```

b

***cst-1* genomic seq annot:**

Lt gray: UTR

Yellow: exons

Green: alternating exons

Purple: exon 9 sequence that is IDENTICAL with *cst-2* exon 9.

```
>Unspliced + UTR + upstream + downstream (8069bp)
attttttcaaaaatatatacacccataatatgaaaaagtattttcaactatcaataaacaccgagaaaagcggaatatcaa
aatttttatgaattaggggtgggaggcaattgttaaaatgcggaaaatattgaattgctgatgaattcaagattcggcga
ttcccgaaaataccgaatattttcaaactcggcaatctggaatagcagacatttagtagggaaacgttgtatgtcttccc
aaaaatatttattacaatgaactattgtttgtttgtttgtttttgaattttatttgttcaaaattatcgattgtcaaatt
gccaaaaaatttgatattccgcttttctcggtgtttattgatagttgaaaatactttttcatattatgggtgtatatat
ttttgaaaaatcaattaacagttctttaaagttaaatagattgaaattaaagaataacttggaaattgtttttgaattta
tgacatctctttgcttgaaaaaatggatgaaacatgctttataaaatcaactagaataattgtccaaactgacaaaaagc
aatcaactactatgcaccataacaaataaagtctactgacaagtaaagatagtaaacatacgtgaatcaggtgatgacta
cccaacaactctcataataaaacagtaccgcaaaacagcaccaggaaggacacagttagcaatacgatattccgcattca
aacatcctgacgtgtgaagggtcaagtcttatgaaacgagcgcagtacacacttcgcactctttcccgtccttttccccc
tttcgcctcgaccacacactgctcaaataacgtagagtgagccgagggcattacgaatgctcgtcgaatgtagcgcctgc
ggtcggttatattaacttctttttttttatcacggtacaacttatctgaaatagagtactgtttttgagttgttctctgg
tctttgatagtttgatagtttcaatgggaatatttaattctcacactttcaaaaaggatatttcaatcttaaatgaaatg
ctaacattgcgaaatagaattgaaaatcatgatacttcaatctctttcatgaaaaataaggttcttctaaatttgtattg
ttttaaaatatgctcaggtctatctcaaagatttcataaattataaaatttattaattagtttttctcggctttttcaac
cattttaccaatgaaatcttaatctaaaaacgttggactttgtcggattactttttttttccaaaagacaattgccgtagt
caattattcattcttcgggccatcggggccacatgggtggaaccccgcagacgtcttttcgctttcaaaacgacgtccaa
atcgattcgcctgtctttctcttctcactcttctcgcctcccttcagaatatgaccatacatgcactgttgacgttctaa
ttgaacaagcagagtgcatgtgcaaccaagagaacgcagagaaacatgcgactaatgtgtcctcaaggataggttttatc
ttcttcttcttctttcttcttctttcttcgatttcttgccccatagttctcgtcggtcggacggacaacatctgctctt
catcttacttatatctcattgggtgttcattcctatactcacacaagatcttacaacactttttttataaatccttcaact
tgtacacagttaaaggtggagtagcgcaagttggaaaattgtttaaaactactcttatggtgtcaaaatgactaaatatt
aaacaaataaaaatgctgaacagtttttgaaaggacgactttcaaaaagtccaacaattggcaaaaactgagtttatttt
caattttcaaaaaaaaatcagataaaatttagaacatctttcagaaactttttgctaaaatatttgattattgtggtgcc
gcatgcatgttttttgagcaatttcagcactgacgttactccacttttaaatttcagcaaagttaatttcattaatttcag
cgtcctcccgATGCCACCGTCTACAGACAGCTCGCGGCGGAATTCGGAAGAGGGCTCAAGgtttgtcagacacaaaacaa
aaaaaaagagttactcttccaaaataaacattctcttttgttttagCGATGGATTTAAATTGGATTCGTCGGCGCTTAAC
AAACCACCAGAAGAAGTTTTCGACATTGTTGGAAAGCTGGGCGAAGGgtttgttatttcaattctatcggattgaaaaat
tccaaattttcagATCATATGGAAGCGTACATAAGGCAATTCACAGAGAGTCAGGTCATGTGTTAGCCATCAAAAAAGTG
CCAGTGGATACTGATCTTCAAGAAATTATCAAGGAAATCAGTATAATGCAACAATGTAAAAGTAAATATGTTGTCAAATA
CTATGGATCGTATTTCAAACATTCGGATCTATGGgtgagattgtttagataaattttaagattttaatgctgtgcccaag
tgggactaaataaaagaaattgaacaaaaattatattcgttttgtgaattagagttaaaaacagtaaagttctgattttt
ccacagaaaaaaatgtagcttaaagtggaaataacattcaaaagtattcattcaaatgcatgccaattgaaattcaaaga
ttttattccctacccctagtcgaaaatcaagaagaaaactggactaacaaaaaaactaatttaaagATTGTGATGGAATA
CTGTGGAGCTGGGTCGATCTCCGATATAATGCGTGCCAGGCGGAAACCGTTATCCGAACAAGAAATCAGTGCAGTTTTGC
GCGATACTCTCAAGGGATTGCAATATTTGCATGACTTGAAGAAGATTCATAGAGATATAAAGgttagaaactgttttttt
ttgaaaaaaaaactgtattttcagGCTGGAAACATTCTACTCAATACCGATGGAATTGCAAAGCTTGCGGATTTCGGTGT
CGCTGGACAGTTGACTGATACAATGGCAAAAAGAAATACAGTCATTGGAACGCCATTCTGGATGGCCCCTGAAGTTATCG
AAGAAATCGGATATGACACAAAAGCTGATATATGGTCACTGGGAATAACAGCTATCGAAATGGCAGAGGGACGGCCTCCT
TATTCAGATATTCATCCGATGCGAGCAATCTTCATGATTCCAACTAAACCACCACCTACGTTTAAAAAACCTGAAGAATG
GTCGTCCGAGTTCAATGATTTCATAAGAAGCTGTTTAATTAAAAAGCCAGAAGAAAGAAAAACGGCACTCCGATTGTGTG
AGgtacgtatggcttaaaaaaaaaagaaatttcccaataagatttatccaaaaaatattgaaattctcaaatattcgttat
atttgttaaaactgtgaaaaaaggtacatccacctaaaattcaaaatttcgacaacttttttttgtcgcagggactagaa
attaattttcaatcatgtagtatttctttttctatttttaaagtttctagtcggtagtcagctaaacttgtgttctaa
tcggctgaaaatatgtccatgtcagcaaaaaatgcgagaaaagcgataaactttcacgtttgactacaaaatgatgtcga
```

ataaaagcttgaaatctataaaaacaaacaatccaaaaaacgctagaactcaacataattctctactcggtattcagagg
caacttcctgccacactttgacaacttatttagtgccactaaaataagttgccaaattgctggcaggaagttgccgagtt
gttggcaggaagttgccacgtagtggcagtaagtgggaaaaaacatctaaatgttggcaggaatgtgcaaaagtttggca
gaaagttgccaaaagttggcaggaagttgcctttaaatgccgaggagcgttctttcccacttgctgccactacgtggcaa
ctcatttaaaaactgccaagatatagacggggccaaattttcatatttcctacaactactctcaaccgaaaatagaagat
ttcttataatttgacaaccccttcggagtaatttttaactttttacatagaattttctactatttttatacacttttttt
gttgttttctgtcatagtattactaatactgctgaaaaacaaatgatacacagtttgtgaacattgctcacttcatagat
atttttcagCACACATTCATCAAAAATGCACCAGGTTGCGATATTATGCAGTTGATGATCCAGGATGCTCAAGAAAAAGC
TATACTAGGACAAGCACCAATGgttgacaaaaaatttcattagaaaacgaaaatttgattgtttttttcagGCAGCAAGCA
GCGGAAATGATGCAACGTTGCTAAGCGAGGGAATGTCCACTATGATTGACGGTGGAGAGTCTACGTTAGTTCAACACAAA
Ggtattatgatagttttgaatatgtaatttgatcatatgtgtcgtgggaaagtgcaaaactcacgcacctaatttgcact
actttgtagctcgatatttagtagtaacgtgaagcaggacacagggagaaattacaaaattacctagtgaaatagaactc
acaatttcagACAACTATGTCACTGCTCAAAGTCTTCGAAGTCAAATGGAAAGCTTGAGAATTGGTGGAGAAATTCCTAA
ATCGGCATATAgttcgtttttttcatgagcgttaaattgcattcaccgatatagttccag**GCTCGTCGAAAAATGGCAACT**
CACCGCGCGTTCAACCACCGGGTCACACCGCAAGCGCTTCAGACCCTTCAAAAAACCAACCGTTTGCTCAAGATGGAACC
GGTCCAAACTTTCAATTGGGCACCAGCGAGAGCAGTTACAAAGATGCTAGTTATAATATGAgttagttatttgaaagaaa
accaattataaaatgatgatctatgagaaaaaaaagctagcataaaaaacgttattcagctcgaatgactttgacaaaaa
aatgaaaaattattcaggcattttcacaaacctaaaattttattaaaattgaaatgtcataccccttattctagccatgc
atgaaatgttagctgattaagattttgcctgtaaattctaaaaaatgaaaaatgtcactactaatgttttgtggaaaatt
tgtattgcataagttttgaaacttagaaagacatattttgaaatcttgaattgaatcacagttaaaaaacacctactctt
tcagTGAACACGGAAGCCGAATACGAAAATAGATTTCAACGGGCAGTGGTCGATGGAGATTTCGAATTTgtaagtgtatt
tcgtaatttttctaataacgaaatagctgcatttagTTACGAAACATCACGCTAGACGAACTGATTCGAAGGAAAGAAAG
TTTGGATTCGGAGATGGAAGAAGAAATACGAGAGTTGCAGAGAAGgtaaaatctttttttttttaattggttgaatttgag
gaaatttaattcaaacacctcaaaaagccggaaattgagattttaacaaaaaatcctaagcgaattttctagctggatt
ttttgtgagtttactgatttaaacatctaaaggacagttttatgaatcgcgaactactgctatttataatttttaagaaa
tagtgatgctgaaattttgctcagaattttttttctttgatgcttaaaattccattctcaaatttaggatatttgaagctt
ttggcatcgaatgttttcaaaatgagccaacttttctctttttttttcttacattttggagtcaagaaaattgatattaaaa
tccgcaacagtggcaattcacgattaaagtcaaatactaaaaaaagaacaaaatttcagATACAAAACAAAAAGGCAACC
AATACTGGATGTTATAGAAATCAAAAAACGACTTCAA**TGA**cggcttcgatacatgcttgtttaataataatttttttgtc
aatctgcaaatatatgaaacctattaattaactagattgtattttaaatgtcatatttattctaaattttgttagatcag
aagcaagagcaaactgaacttgttgtgttttgcttagctttttcatgctaaaataaatctggtcaattttaattttttg
ggaaggtttgttctgaaggcggatattttatcagtgactaacaaggaacaatgggagtgacggatataatttttcgaata
acggaaatagataaaatttcacttacaattgaaaaagctgcaatgagtcagagcgatagatggttgatctcgatgagtct
agtttttcttatttgtttgcctaccgcggagttcacgtagcgaccaattaatattttaggtaaattttgcatggcatcc
tagtagaaacaaaaaaccacattctgagtgcttttttgcattacgactgccgttttgaatcgtaagatttatggcttatga
cggacgtccttttagctttagtctgttataattgtcatattgggtctctgctgccgttgggaagcagatatatttcgtca
actactagccgagagggcgtttgctttttttttttcaagaagaggcaaatatacacttcgaggcgaatgtttctcaactg
tcaggtaatatttgcgaaaccgaaatatatttaaggagtgaaggaagcgaagattgccatcgaacaaaagttcatccgct
atcagcttatcaacaatgttcgcacttattctttctttgtgtctttttcaaggtaaactattctttcagaattatagtat
tttctgaaaaatgtttccaatttctgaaacagttttttttgattgttataaaatattttaggaacactttcatatgta
attggaggcggtgtacaagcaaatccaagcggatgcaacgtatgtggtgatagccctcagtggagtggatggacagaatg
gtcttcctgttctgccgcttttggaagtaggtcttctatttttgaatttttgctatagcagttttgcgaaaacctgaca
catcaatttaaagaaacattcagttttactggtcccgccacaatttcagtgatctttatgaaatgtaaaattctacagtg
cacagagattcttggatttgaaatagaatttccaacacctggagtcacatttccgctatatgtacatcttcataataata
atttcaatttcagcgccagtccaaactcgcactagactttgtccatccggcaactgccaaggaggttctacaacagaatc
aaaaccatgcgtcctttatgatccacaaccaactcaaccacaatggggagcttggggaggttggagtagttgcaggttag
caatattttgctttaatctgtttattattgttttgttttctttattgccaaactgataagcctaactttttctttttgcag
tgccacctgcggcggtggtacgatgacgagaagtcgtgtttgcaataacggatgctcaacctgccaatgtgtcggagctg
ctgccgagtcgcaagcttgcaatgctcaaccgtgctgcacctggactgctgtgagaacaaattgagttcaacttctcact
taataactttgtttcagtggtcttcctggtcggcgtgctcagtaacctgcggatctggaggatcaatcaccagatctag
gcaatgtagctgtggatcgggagtaagtttaaaatttataaagctaaacgtttttattcaatataacatttataactgtt
tgtttcagtgtactggaggctccgttgaacaagagccatgccctcaacaagctgcttgcccgtgcacaacatgcaatcaa
ccaccaccaccatgcaatacttgtaatacccaaccggttgtcattgtgactccagctccatgcacaacttgttaccaacc
accagcgtgctcaacctgcgggcatgcacaacctttctatgatccatacggaaatggaagaaagaagagaatgattacag
tatctggaaattcgacaagtgcttaactgtattgaaacttgtgtacatttatgcaatattttgcacattccgaagcaata
taaataaagtattgagcaattattatttctatccactacagcagaatcacagaaccactaaaaaaacaaa

C

***cst-2* genomic seq annot:**

Lt gray: UTR

Yellow: alternating exons

Green: alternating exons

Purple: exon 9 sequence that is IDENTICAL with *cst-1* exon 9.

Pink: start of next gene downstream, unrelated

>Unspliced + UTR + upstream + downstream (6994bp)
taattgattttcaaaaatatatacacccataatatgaaaaagtattttcaactatcaataaacaccgagaaaagcggaa
tatcaaaatttttggcaatttgacaatcgataattttgaacaaataaaattcaaaaacaaacaaacaaacaatagttca
ttgtaataaatatttttgggaagacatacaacgtttccctactaaatgtctgctattccagattgccgagtttgaaaata
ttcggtattttcgggaatcgccgaatcttgaattcatcagcaattcaatattttccgcattttaacaattgcctcccacc
cctaattcataaaaatttttgatattccgcttttctcggtgtttattgatagttgaaaatactttttcatattatgggtgt
atatattttgaaaaatcaattaacagttctttaaagttaaatagattgaaattaaagaataacttggaaattgtttttg
aatttatgacatctctttgcttgaaaaaatggatgaaacatgctttataaaatcaactagaataattgtccaaactgaca
aaaagcaatcaactactatgcaccataacaaataaagtctactgacaagtaaagatagtaaacatacgtgaatcaggtga
tgactacccaacaactctcataataaaacagtaccgcaaaacagcaccaggaaggacacagttagcaatacgatattccg
cattcaaacatcctgacgtgtgaagggtcaagtcttatgaaacgagcgcagtacacacttcgcactctttcccgtccttt
tccccctttcgcctcgaccacacactgctcaaataacgtagagtgagccgagggcattacgaatgctcgtcgaatgtagc
gcctgcggtcggttatattaacttcttttttttttatcacggtacaacttatctgaaatagagtactgtttttgagttgtt
ctctggtctttgatagtttgatagtttcaatgggaatatttaattctcacactttcaaaaaggatatttcaatcttaaat
gaaatgctaacattgcgaaatagaattgaaaatcatgatacttcaatctctttcatgaaaaataaggttcttctaaattt
gtattgttttaaaatatgctcaggtctatctcaaagatttcataaattataaaatttattaattagtttttctcggcttt
ttcaaccattttaccaatgaaatcttaatctaaaaacgttggactttgtcggattactttttttttccaaaagacaattgc
cgtagtcaattattcattcttcgggccatcggggccacatgggtggaaccccgcagacgtcttttcgctttcaaaacgac
gtccaaatcgattcgcctgtctttctcttctcactcttctcgcctcccttcagaatatgaccatacatgcactgttgacg
ttctaattgaacaagcagagtgcatgtgcaaccaagagaacgcagagaaacatgcgactaatgtgtcctcaaggataggt
tttatcttcttcttcttctttcttcttctttcttcgattttcttgccccatagttctcgtcggtcggacggacaacatct
gctcttcatcttacttatatctcattgggtgttcattcctatactcacacaagatcttacaacactttttttataaatcct
tcaacttgtacacagttaaaggtggagtagcgcaagttggaaaattgtttaaaactactcttatggtgtcaaaatgacta
aatattaaacaaataaaaatgctgaacagtttttgaaaggacgactttcaaaaagtccaacaattggcaaaaactgagtt
tattttcaattttcaaaaaaaaaatcagataaaatttagaacatctttcagaaactttttgctaaaatatttgattattgt
ggtgccgcatgcatgtttttgagcaatttcagcactgacgttactccacttttaaatttcagcaaagttaatttcattaa
tttcagcgtcctcccg**ATG**CCACCGTCTACAGACAGCTCGCGGCGGAATTCGGAAGAGGGCTCAAGgtttgtcagacaca
aaacaaaaaaaaagagttactcttccaaaataaacattctcttttgttttagCGATGGATTTAAATTGGATTCGTCGGCG
CTTAACAAACCACCAGAAGAAGTTTTCGACATTGTTGGAAAGCTGGGCGAAGGgtttgttatttcaattctatcggattg
aaaaattccaaattttcagATCATATGGAAGCGTACATAAGGCAATTCACAGAGAGTCAGGTCATGTGTTAGCCATCAAA
AAAGTGCCAGTGGATACTGATCTTCAAGAAATTATCAAGGAAATCAGTATAATGCAACAATGTAAAAGTAAATATGTTGT
CAAATACTATGGATCGTATTTCAAACATTCGGATCTATGGgtgagattgtttagataaattttaagattttaatgctgtg
cccaagtgggactaaataaaagaaattgaacaaaaattatattcgttttgtgaattagagttaaaaacagtaaagttctg
atttttccacagaaaaaaatgtagcttaaagtggaaataacattcaaaagtattcattcaaatgcatgccaattgaaatt
caaagattttattccctacccctagtcgaaaatcaagaagaaaactggactaacaaaaaaactaatttaaagATTGTGAT
GGAATACTGTGGAGCTGGGTCGATCTCCGATATAATGCGTGCCAGGCGGAAACCGTTATCCGAACAAGAAATCAGTGCAG
TTTTGCGCGATACTCTCAAGGGATTGCAATATTTGCATGACTTGAAGAAGATTCATAGAGATATAAAGgttagaaactgt
ttttttttgaaaaaaaaactgtattttcagGCTGGAAACATTCTACTCAATACCGATGGAATTGCAAAGCTTGCGGATTT
CGGTGTCGCTGGACAGTTGACTGATACAATGGCAAAAAGAAATACAGTCATTGGAACGCCATTCTGGATGGCCCCTGAAG
TTATCGAAGAAATCGGATATGACACAAAAGCTGATATATGGTCACTGGGAATAACAGCTATCGAAATGGCAGAGGGACGG
CCTCCTTATTCAGATATTCATCCGATGCGAGCAATCTTCATGATTCCAACTAAACCACCACCTACGTTTAAAAAACCTGA
AGAATGGTCGTCCGAGTTCAATGATTTCATAAGAAGCTGTTTAATTAAAAAGCCAGAAGAAAGAAAAACGGCACTCCGAT
TGTGTGAGgtacgtatggcttaaaaaaaaagaaatttcccaataagatttatccaaaaaatattgaaattctcaaatatt
cgttatatttgttaaaactgtgaaaaaaggtacatccacctaaaattcaaaatttcgacaacttttttttgtcgcaggga
ctagaaattaattttcaatcatgtagtatttcttttttctattttttaaagtttctagtcggtagtcagctaaacttgtg
ttctaatcggctgaaaatatgtccatgtcagcaaaaaatgcgagaaaagcgataaactttcacgtttgactacaaaatga
tgtcgataaaagcttgaaatctataaaaacaaacaatccaaaaaacgctagaactcaacataattctctactcggtattc

c

agaggcaacttcctgccacactttgacaacttatttagtgccactaaaataagttgccaaattgctggcaggaagttgcc
gagttgttggcaggaagttgccacgtagtggcagtaagtgggaaaaaacatctaaatgttggcaggaatgtgcaaaagtt
tggcagaaagttgccaaaagttggcaggaagttgcctttaaatgccgaggagcgttctttcccacttgctgccactacgt
ggcaactcatttaaaaactgccaagatatagacggggccaaattttcatatttcctacaactactctcaaccgaaaatag
aagatttcttataatttgacaaccccttcggagtaatttttaactttttacatagaattttctactattttttatacact
tttttgttgttttctgtcatagtattactaatactgctgaaaaacaaatgatacacagtttgtgaacattgctcacttca
tagatatttttcagCACACATTCATCAAAAATGCACCAGGTTGCGATATTATGCAGTTGATGATCCAGGATGCTCAAGAA
AAAGCTATACTAGGACAAGCACCAATGgttgacaaaaatttcattagaaaacgaaaatttgattgtttttcagGCAGC
AAGCAGCGGAAATGATGCAACGTTGCTAAGCGAGGGAATGTCCACTATGATTGACGGTGGAGAGTCTACGTTAGTTCAAC
ACAAAGgtattatgatagttttgaatatgtaatttgatcatatgtgtcgtgggaaagtgcaaaactcacgcacctaattt
gcactactttgtagctcgatatttagtagtaacgtgaagcaggacacagggagaaattacaaaattacctagtgaaatag
aactcacaatttcagACAACTATGTCACTGCTCAAAGTCTTCGAAGTCAAATGGAAAGCTTGAGAATTGGTGGAGAAATT
CCTAAATCGGCATATAgttcgtttttcatgagcgttaaattgcattcaccgatatagttccag**GCTCGTCGAAAAATGG**
CAACTCACCGCGCGTTCAACCACCGGGTCACACCGCAAGCGCTTCAGACCCTTCAAAAAACCAACCGTTTGCTCAAGATG
GAACCGGTCCAAACTTTCATCCACTATCTCAACACTTTGTTTTGGTTTTTATAATT**TAA**aagcctagctaatatcctaat
ctgtttacctgatataatagttttcaaatttaccttctggaaaacttatatattactatactgtcgaaacggatgccgca
catccctttgctgtaaaaattgccgagttcctaactctttgtccgcaaaactcacaaatagtctatgagataatgcatgt
tctcttctgaacctttcaaatcaactatagctggtaagtactctttcaattagttcagcttcttttcttttctcaccaac
tactatctcgactccgacctatttagatcggaaccaattctctcccgatcacttcaacatatgaaaaagacgaatttgtg
agttagagagtatactcgaaatagtaacattgatttccccatttttcgactttcttttcttcgctatcgtgtgtactta
tcgcatgtaacatttgctgattagctgactgaccacggcttcaagtagcaaatttatttctagcacgtgaggttgcatag
atgagtaccttcgttagctttgctttcaggaactcaaaattgaaaagagaaacatgtggtgaagcagcggtaggtgaaac
tctttttctaagctattgcaagattttctcaagatcacacgcctagtatgcagggtgagaatgccggatgcattgaaaat
aaatgcaacaagttgcagctatgattagctttttttaatgattcgactgctttcagaatggctttatattgcaccagctc
tttgagggatctttgaataatttttttcaggttgaaaagcgaaag**atg**agacgtttatccgagatgatatctgattggga
acgaccgtcaagaagttcgatcagtaacatgtatctttatgcgagaagagaaagcgtctacattgcaaggaagaagttgg
ccggtctgaaagtaataatttgagctttaaaaacaaacacactataaagactgaaaataatccgtttttaatacaaaact
gtttaaaaaaatttaaaaaagattcatacttgttgttgttctagaatgttcatggttcaatttagattagctcaagtatg
attttaaatatttttttgttatatccagattgaaaatacgtagaatattcaaagaaagacattgaaatttgaattacctgc
aaaatacgcatgtttcagccacttttggtaactcaacgctaaattttcataaaaattttccatatcctttgaaacatccg
gaatctaaaaaaaaacaagaaaaaaacggaaaatattggaactataccaaaataaaattaccgaattttcttcaactgtc
cgattttccaggactgggcgctggacctgttagcaaaactgaaatccaagcagggaatggcgattcgtcgagagactcgt
gcaaccaagttggttgcaactgtaatgggtaactgaaactattctttgacattaaaactaaaccaaatacattgcagtgg
tttttctcgtctgctggcttccgtttttcacattgaacatgatcaaaatttacaaattgatattcaacgtttggtcagcg
gacctcgaaatttggttccattggtttaccgcactggggtatttgaattcgtcactcaactttttcatttactcaacaat
caatccagtaagcattttttatatcaaatttgatagtaattatagaaataattataatacataattggctatataataaaa
aagtttagtcgaaaaagattatatttttaatttttcaagttcaatttgcgttgtataaaacattaattcatggaattgag
acaaggtaaaaccgacaacgttttccaaaatttgacatgtctgtattccctctataggcattgcgccagcttaactcaa
tttgacaagaaatagtaaacccgcttagcaaactaaatatactgaaaactgtactgtataatccattcaacacctccttt
tttcagaaattccgtcattcattccgccgcttacttggcttccgtcgatcttctcgacgtcagagagaaaaatcgtggat
gttaccgccacgagatagcagcaggtgagcctcatttattcgtccatagcttttaaatgtttcataacagaggtcctgca
aaatgtggatgcggtttccataaaatcgtgaaaa
