# Supplementary material for: Canonical and noncanonical Hippo signaling in C. elegans
Source: Genetics. 2026 Feb 26;233(1):iyag056. doi: 10.1093/genetics/iyag056 (PMC13147543; doi:10.1093/genetics/iyag056)
Supplement: iyag056_Supplementary_Data [file iyag056_supplementary_data.zip › Figure_S1_GENETICS-2025-308930.pptx]

## Slide 1
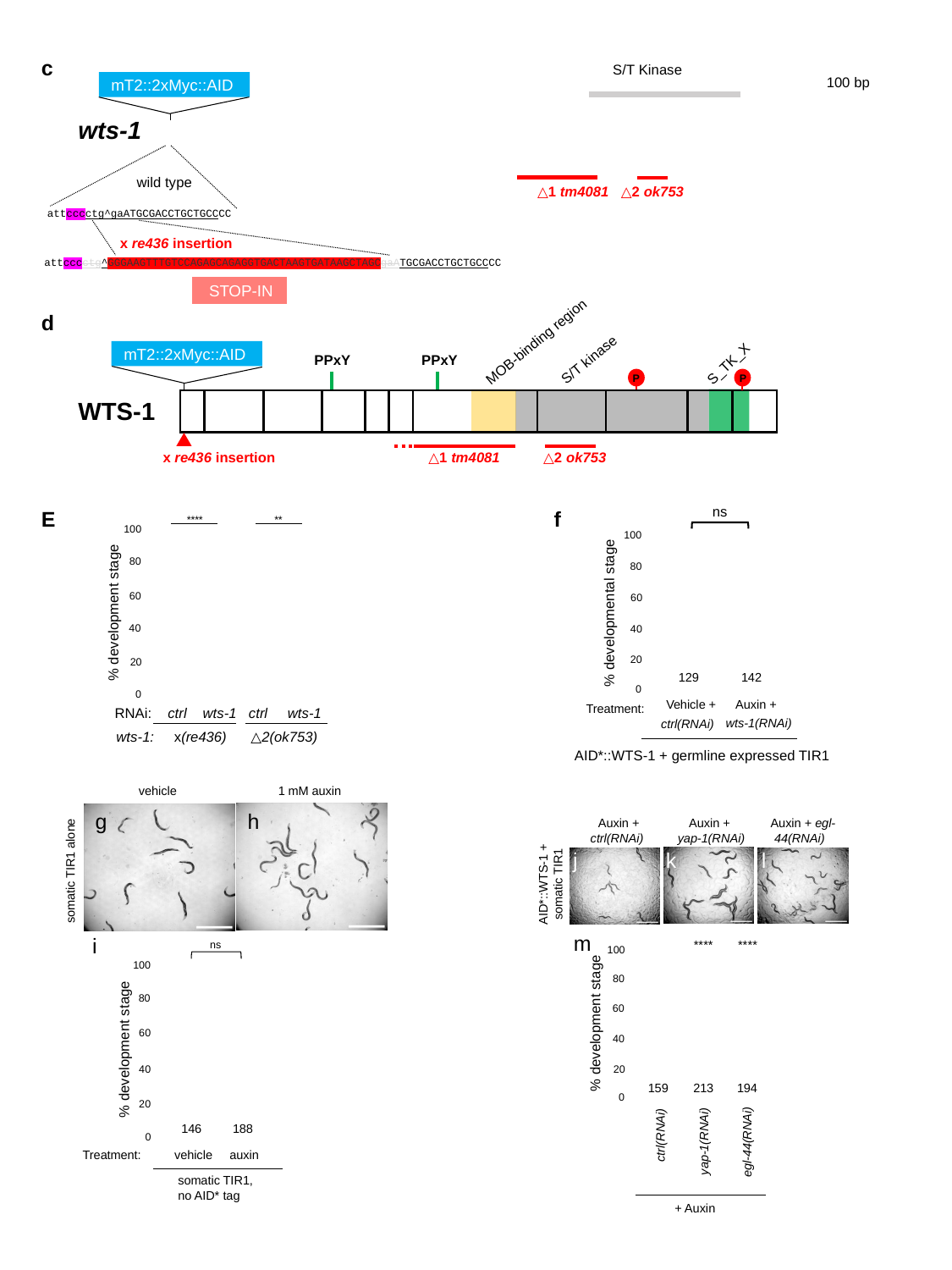

c
S/T Kinase
100 bp
 mT2::2xMyc::AID*
wts-1
wild type
△1 tm4081
△2 ok753
attcccctg^gaATGCGACCTGCTGCCCC
 x re436 insertion
attcccctg^GGGAAGTTTGTCCAGAGCAGAGGTGACTAAGTGATAAGCTAGCgaATGCGACCTGCTGCCCC
 STOP-IN
d
S/T kinase
MOB-binding region
 mT2::2xMyc::AID*
S_TK_X
PPxY
PPxY
P
P
WTS-1
 x re436 insertion
△1 tm4081
△2 ok753
ns
100
80
60
% developmental stage
40
20
 0
Auxin +
Vehicle +
Treatment:
wts-1(RNAi)
ctrl(RNAi)
AID*::WTS-1 + germline expressed TIR1
E
f
****
**
100
80
60
% development stage
40
20
129
142
97
80
106
92
0
RNAi: ctrl wts-1 ctrl wts-1
wts-1: x(re436) △2(ok753)
vehicle
1 mM auxin
h
g
somatic TIR1 alone
i
ns
100
80
60
% development stage
40
20
146
188
0
Treatment:
vehicle
auxin
somatic TIR1, no AID* tag
Auxin + ctrl(RNAi)
Auxin +
yap-1(RNAi)
 Auxin + egl-44(RNAi)
j
k
l
AID*::WTS-1 + somatic TIR1
m
****
****
100
 80
 60
% development stage
 40
 20
159
213
194
 0
ctrl(RNAi)
yap-1(RNAi)
egl-44(RNAi)
+ Auxin

## Slide 2
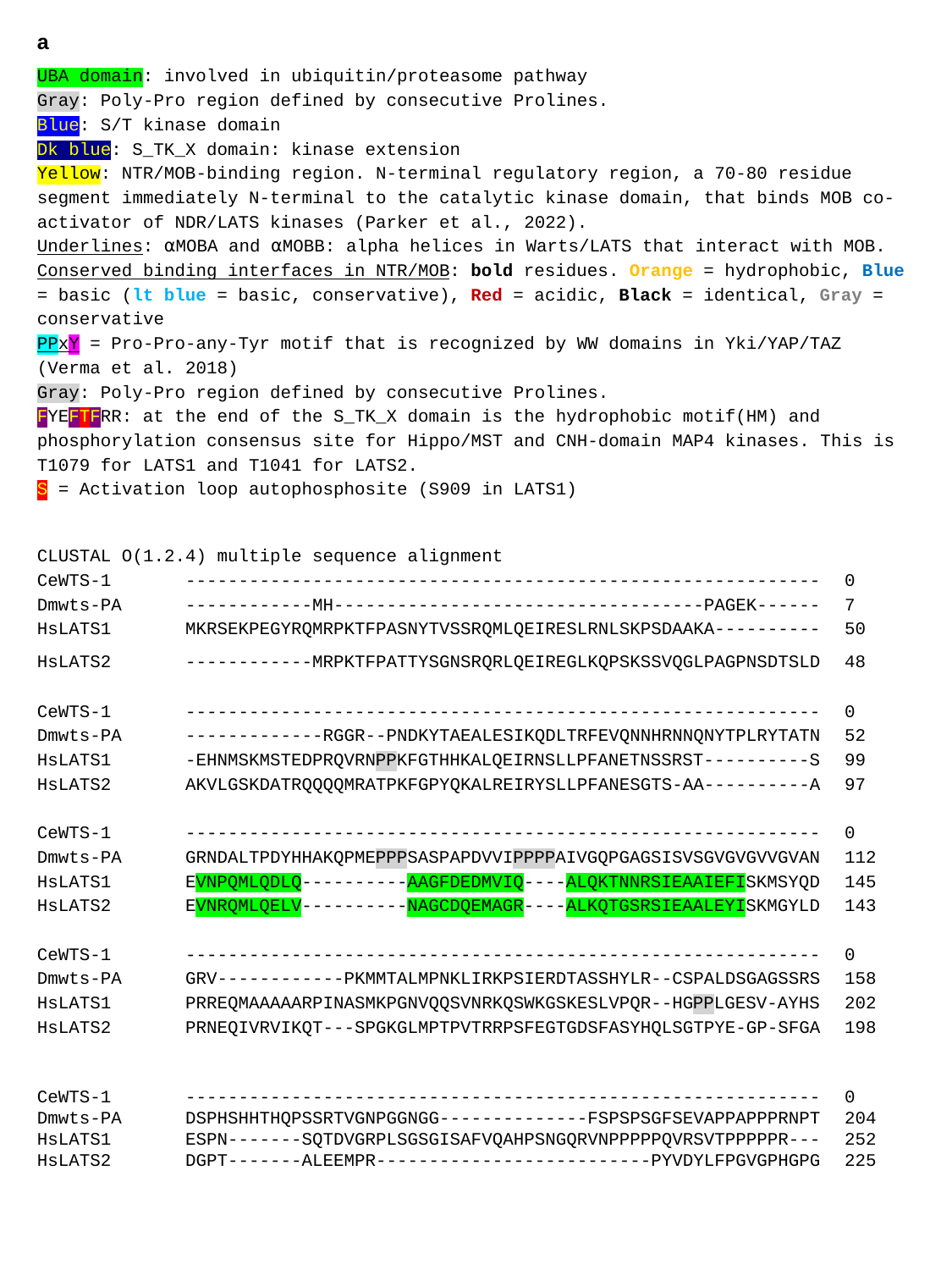

UBA domain: involved in ubiquitin/proteasome pathway
Gray: Poly-Pro region defined by consecutive Prolines.
Blue: S/T kinase domain
Dk blue: S_TK_X domain: kinase extension
Yellow: NTR/MOB-binding region. N-terminal regulatory region, a 70-80 residue segment immediately N-terminal to the catalytic kinase domain, that binds MOB co-activator of NDR/LATS kinases (Parker et al., 2022).
Underlines: ⍺MOBA and ⍺MOBB: alpha helices in Warts/LATS that interact with MOB.
Conserved binding interfaces in NTR/MOB: bold residues. Orange = hydrophobic, Blue = basic (lt blue = basic, conservative), Red = acidic, Black = identical, Gray = conservative
PPxY = Pro-Pro-any-Tyr motif that is recognized by WW domains in Yki/YAP/TAZ (Verma et al. 2018)
Gray: Poly-Pro region defined by consecutive Prolines.
FYEFTFRR: at the end of the S_TK_X domain is the hydrophobic motif(HM) and phosphorylation consensus site for Hippo/MST and CNH-domain MAP4 kinases. This is T1079 for LATS1 and T1041 for LATS2.
S = Activation loop autophosphosite (S909 in LATS1)
CLUSTAL O(1.2.4) multiple sequence alignment
CeWTS-1 ------------------------------------------------------------	0
Dmwts-PA ------------MH-----------------------------------PAGEK------	7
HsLATS1 MKRSEKPEGYRQMRPKTFPASNYTVSSRQMLQEIRESLRNLSKPSDAAKA----------	50
HsLATS2 ------------MRPKTFPATTYSGNSRQRLQEIREGLKQPSKSSVQGLPAGPNSDTSLD	48
CeWTS-1 ------------------------------------------------------------	0
Dmwts-PA -------------RGGR--PNDKYTAEALESIKQDLTRFEVQNNHRNNQNYTPLRYTATN	52
HsLATS1 -EHNMSKMSTEDPRQVRNPPKFGTHHKALQEIRNSLLPFANETNSSRST----------S	99
HsLATS2 AKVLGSKDATRQQQQMRATPKFGPYQKALREIRYSLLPFANESGTS-AA----------A	97
CeWTS-1 ------------------------------------------------------------	0
Dmwts-PA GRNDALTPDYHHAKQPMEPPPSASPAPDVVIPPPPAIVGQPGAGSISVSGVGVGVVGVAN	112
HsLATS1 EVNPQMLQDLQ----------AAGFDEDMVIQ----ALQKTNNRSIEAAIEFISKMSYQD	145
HsLATS2 EVNRQMLQELV----------NAGCDQEMAGR----ALKQTGSRSIEAALEYISKMGYLD	143
CeWTS-1 ------------------------------------------------------------	0
Dmwts-PA GRV------------PKMMTALMPNKLIRKPSIERDTASSHYLR--CSPALDSGAGSSRS	158
HsLATS1 PRREQMAAAAARPINASMKPGNVQQSVNRKQSWKGSKESLVPQR--HGPPLGESV-AYHS	202
HsLATS2 PRNEQIVRVIKQT---SPGKGLMPTPVTRRPSFEGTGDSFASYHQLSGTPYE-GP-SFGA	198
CeWTS-1 ------------------------------------------------------------	0
Dmwts-PA DSPHSHHTHQPSSRTVGNPGGNGG--------------FSPSPSGFSEVAPPAPPPRNPT	204
HsLATS1 ESPN-------SQTDVGRPLSGSGISAFVQAHPSNGQRVNPPPPPQVRSVTPPPPPR---	252
HsLATS2 DGPT-------ALEEMPR--------------------------PYVDYLFPGVGPHGPG	225
a

## Slide 3
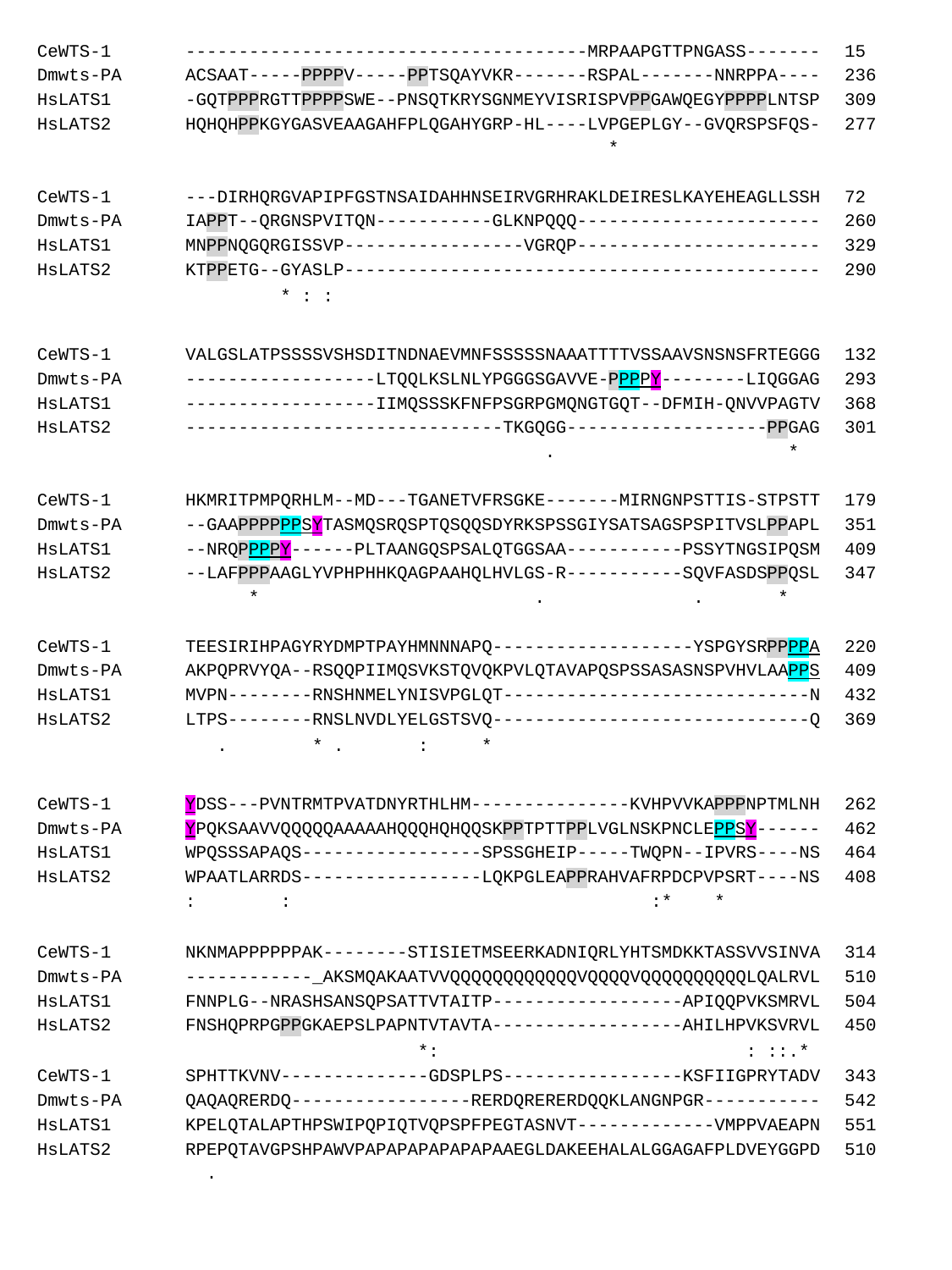

CeWTS-1 --------------------------------------MRPAAPGTTPNGASS-------	15
Dmwts-PA ACSAAT-----PPPPV-----PPTSQAYVKR-------RSPAL-------NNRPPA----	236
HsLATS1 -GQTPPPRGTTPPPPSWE--PNSQTKRYSGNMEYVISRISPVPPGAWQEGYPPPPLNTSP	309
HsLATS2 HQHQHPPKGYGASVEAAGAHFPLQGAHYGRP-HL----LVPGEPLGY--GVQRSPSFQS-	277
 *
CeWTS-1 ---DIRHQRGVAPIPFGSTNSAIDAHHNSEIRVGRHRAKLDEIRESLKAYEHEAGLLSSH	72
Dmwts-PA IAPPT--QRGNSPVITQN-----------GLKNPQQQ-----------------------	260
HsLATS1 MNPPNQGQRGISSVP-----------------VGRQP-----------------------	329
HsLATS2 KTPPETG--GYASLP---------------------------------------------	290
 * : :
CeWTS-1 VALGSLATPSSSSVSHSDITNDNAEVMNFSSSSSNAAATTTTVSSAAVSNSNSFRTEGGG	132
Dmwts-PA ------------------LTQQLKSLNLYPGGGSGAVVE-PPPPY--------LIQGGAG	293
HsLATS1 ------------------IIMQSSSKFNFPSGRPGMQNGTGQT--DFMIH-QNVVPAGTV	368
HsLATS2 ------------------------------TKGQGG-------------------PPGAG	301
 . *
CeWTS-1 HKMRITPMPQRHLM--MD---TGANETVFRSGKE-------MIRNGNPSTTIS-STPSTT	179
Dmwts-PA --GAAPPPPPPSYTASMQSRQSPTQSQQSDYRKSPSSGIYSATSAGSPSPITVSLPPAPL	351
HsLATS1 --NRQPPPPY------PLTAANGQSPSALQTGGSAA-----------PSSYTNGSIPQSM	409
HsLATS2 --LAFPPPAAGLYVPHPHHKQAGPAAHQLHVLGS-R-----------SQVFASDSPPQSL	347
 * . . *
CeWTS-1 TEESIRIHPAGYRYDMPTPAYHMNNNAPQ-------------------YSPGYSRPPPPA	220
Dmwts-PA AKPQPRVYQA--RSQQPIIMQSVKSTQVQKPVLQTAVAPQSPSSASASNSPVHVLAAPPS	409
HsLATS1 MVPN--------RNSHNMELYNISVPGLQT-----------------------------N	432
HsLATS2 LTPS--------RNSLNVDLYELGSTSVQ------------------------------Q	369
 . * . : *
CeWTS-1 YDSS---PVNTRMTPVATDNYRTHLHM---------------KVHPVVKAPPPNPTMLNH	262
Dmwts-PA YPQKSAAVVQQQQQAAAAAHQQQHQHQQSKPPTPTTPPLVGLNSKPNCLEPPSY------	462
HsLATS1 WPQSSSAPAQS-----------------SPSSGHEIP-----TWQPN--IPVRS----NS	464
HsLATS2 WPAATLARRDS-----------------LQKPGLEAPPRAHVAFRPDCPVPSRT----NS	408
 : : :* *
CeWTS-1 NKNMAPPPPPPAK--------STISIETMSEERKADNIQRLYHTSMDKKTASSVVSINVA	314
Dmwts-PA ------------_AKSMQAKAATVVQQQQQQQQQQQQVQQQQVQQQQQQQQQQLQALRVL	510
HsLATS1 FNNPLG--NRASHSANSQPSATTVTAITP------------------APIQQPVKSMRVL	504
HsLATS2 FNSHQPRPGPPGKAEPSLPAPNTVTAVTA------------------AHILHPVKSVRVL	450
 *: : ::.*
CeWTS-1 SPHTTKVNV--------------GDSPLPS-----------------KSFIIGPRYTADV	343
Dmwts-PA QAQAQRERDQ-----------------RERDQRERERDQQKLANGNPGR-----------	542
HsLATS1 KPELQTALAPTHPSWIPQPIQTVQPSPFPEGTASNVT-------------VMPPVAEAPN	551
HsLATS2 RPEPQTAVGPSHPAWVPAPAPAPAPAPAPAAEGLDAKEEHALALGGAGAFPLDVEYGGPD	510
 .

## Slide 4
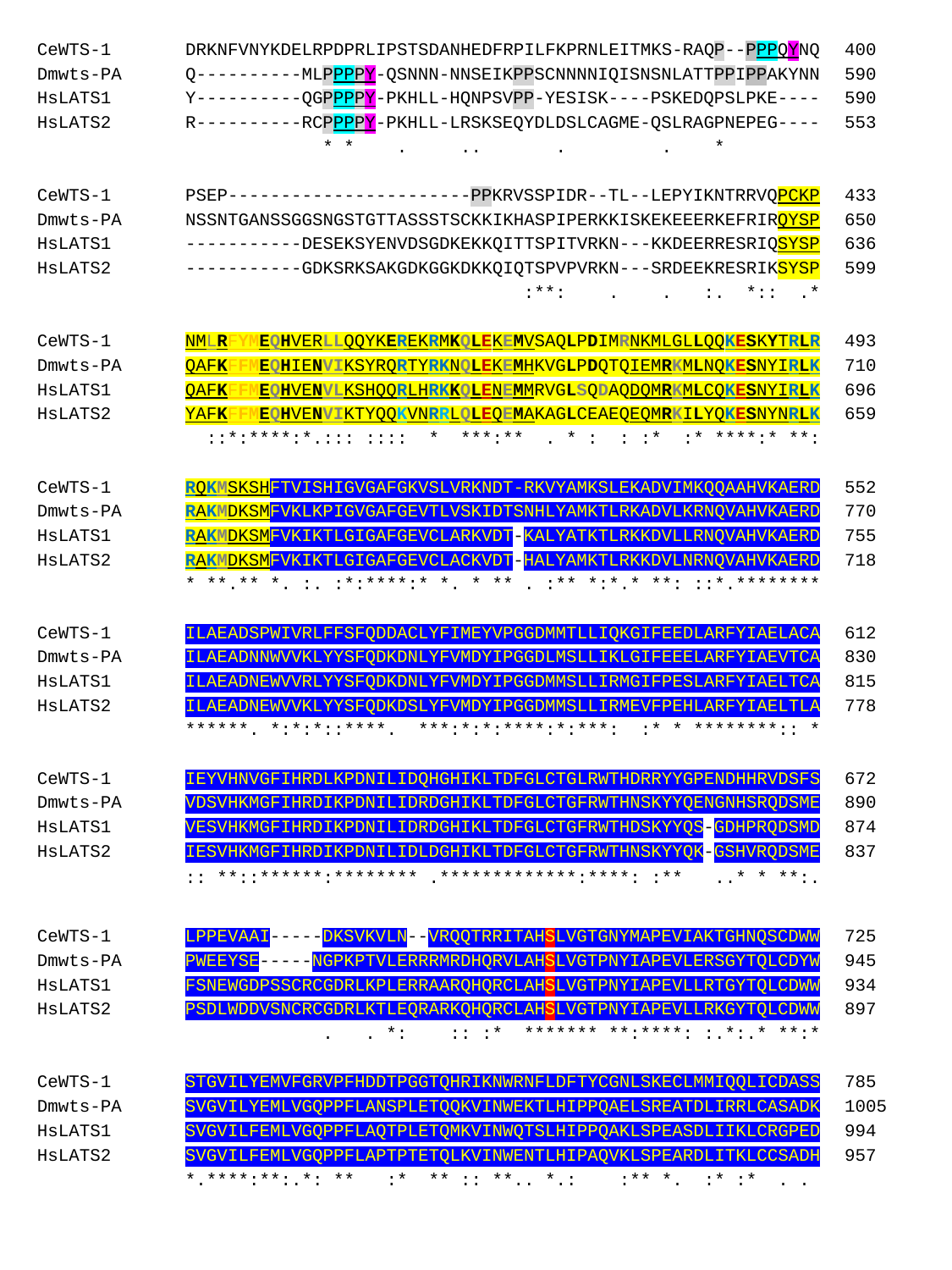

CeWTS-1 DRKNFVNYKDELRPDPRLIPSTSDANHEDFRPILFKPRNLEITMKS-RAQP--PPPQYNQ	400
Dmwts-PA Q----------MLPPPPY-QSNNN-NNSEIKPPSCNNNNIQISNSNLATTPPIPPAKYNN	590
HsLATS1 Y----------QGPPPPY-PKHLL-HQNPSVPP-YESISK----PSKEDQPSLPKE----	590
HsLATS2 R----------RCPPPPY-PKHLL-LRSKSEQYDLDSLCAGME-QSLRAGPNEPEG----	553
 * * . .. . . *
CeWTS-1 PSEP-----------------------PPKRVSSPIDR--TL--LEPYIKNTRRVQPCKP	433
Dmwts-PA NSSNTGANSSGGSNGSTGTTASSSTSCKKIKHASPIPERKKISKEKEEERKEFRIRQYSP	650
HsLATS1 -----------DESEKSYENVDSGDKEKKQITTSPITVRKN---KKDEERRESRIQSYSP	636
HsLATS2 -----------GDKSRKSAKGDKGGKDKKQIQTSPVPVRKN---SRDEEKRESRIKSYSP	599
 :**: . . :. *:: .*
CeWTS-1 NMLRFYMEQHVERLLQQYKEREKRMKQLEKEMVSAQLPDIMRNKMLGLLQQKESKYTRLR	493
Dmwts-PA QAFKFFMEQHIENVIKSYRQRTYRKNQLEKEMHKVGLPDQTQIEMRKMLNQKESNYIRLK	710
HsLATS1 QAFKFFMEQHVENVLKSHQQRLHRKKQLENEMMRVGLSQDAQDQMRKMLCQKESNYIRLK	696
HsLATS2 YAFKFFMEQHVENVIKTYQQKVNRRLQLEQEMAKAGLCEAEQEQMRKILYQKESNYNRLK	659
 ::*:****:*.::: :::: * ***:** . * : : :* :* ****:* **:
CeWTS-1 RQKMSKSHFTVISHIGVGAFGKVSLVRKNDT-RKVYAMKSLEKADVIMKQQAAHVKAERD	552
Dmwts-PA RAKMDKSMFVKLKPIGVGAFGEVTLVSKIDTSNHLYAMKTLRKADVLKRNQVAHVKAERD	770
HsLATS1 RAKMDKSMFVKIKTLGIGAFGEVCLARKVDT-KALYATKTLRKKDVLLRNQVAHVKAERD	755
HsLATS2 RAKMDKSMFVKIKTLGIGAFGEVCLACKVDT-HALYAMKTLRKKDVLNRNQVAHVKAERD	718
 * **.** *. :. :*:****:* *. * ** . :** *:*.* **: ::*.********
CeWTS-1 ILAEADSPWIVRLFFSFQDDACLYFIMEYVPGGDMMTLLIQKGIFEEDLARFYIAELACA	612
Dmwts-PA ILAEADNNWVVKLYYSFQDKDNLYFVMDYIPGGDLMSLLIKLGIFEEELARFYIAEVTCA	830
HsLATS1 ILAEADNEWVVRLYYSFQDKDNLYFVMDYIPGGDMMSLLIRMGIFPESLARFYIAELTCA	815
HsLATS2 ILAEADNEWVVKLYYSFQDKDSLYFVMDYIPGGDMMSLLIRMEVFPEHLARFYIAELTLA	778
 ******. *:*:*::****. ***:*:*:****:*:***: :* * ********:: *
CeWTS-1 IEYVHNVGFIHRDLKPDNILIDQHGHIKLTDFGLCTGLRWTHDRRYYGPENDHHRVDSFS	672
Dmwts-PA VDSVHKMGFIHRDIKPDNILIDRDGHIKLTDFGLCTGFRWTHNSKYYQENGNHSRQDSME	890
HsLATS1 VESVHKMGFIHRDIKPDNILIDRDGHIKLTDFGLCTGFRWTHDSKYYQS-GDHPRQDSMD	874
HsLATS2 IESVHKMGFIHRDIKPDNILIDLDGHIKLTDFGLCTGFRWTHNSKYYQK-GSHVRQDSME	837
 :: **::******:******** .*************:****: :** ..* * **:.
CeWTS-1 LPPEVAAI-----DKSVKVLN--VRQQTRRITAHSLVGTGNYMAPEVIAKTGHNQSCDWW	725
Dmwts-PA PWEEYSE-----NGPKPTVLERRRMRDHQRVLAHSLVGTPNYIAPEVLERSGYTQLCDYW	945
HsLATS1 FSNEWGDPSSCRCGDRLKPLERRAARQHQRCLAHSLVGTPNYIAPEVLLRTGYTQLCDWW	934
HsLATS2 PSDLWDDVSNCRCGDRLKTLEQRARKQHQRCLAHSLVGTPNYIAPEVLLRKGYTQLCDWW	897
 . . *: :: :* ******* **:****: :.*:.* **:*
CeWTS-1 STGVILYEMVFGRVPFHDDTPGGTQHRIKNWRNFLDFTYCGNLSKECLMMIQQLICDASS	785
Dmwts-PA SVGVILYEMLVGQPPFLANSPLETQQKVINWEKTLHIPPQAELSREATDLIRRLCASADK	1005
HsLATS1 SVGVILFEMLVGQPPFLAQTPLETQMKVINWQTSLHIPPQAKLSPEASDLIIKLCRGPED	994
HsLATS2 SVGVILFEMLVGQPPFLAPTPTETQLKVINWENTLHIPAQVKLSPEARDLITKLCCSADH	957
 *.****:**:.*: ** :* ** :: **.. *.: :** *. :* :* . .

## Slide 5
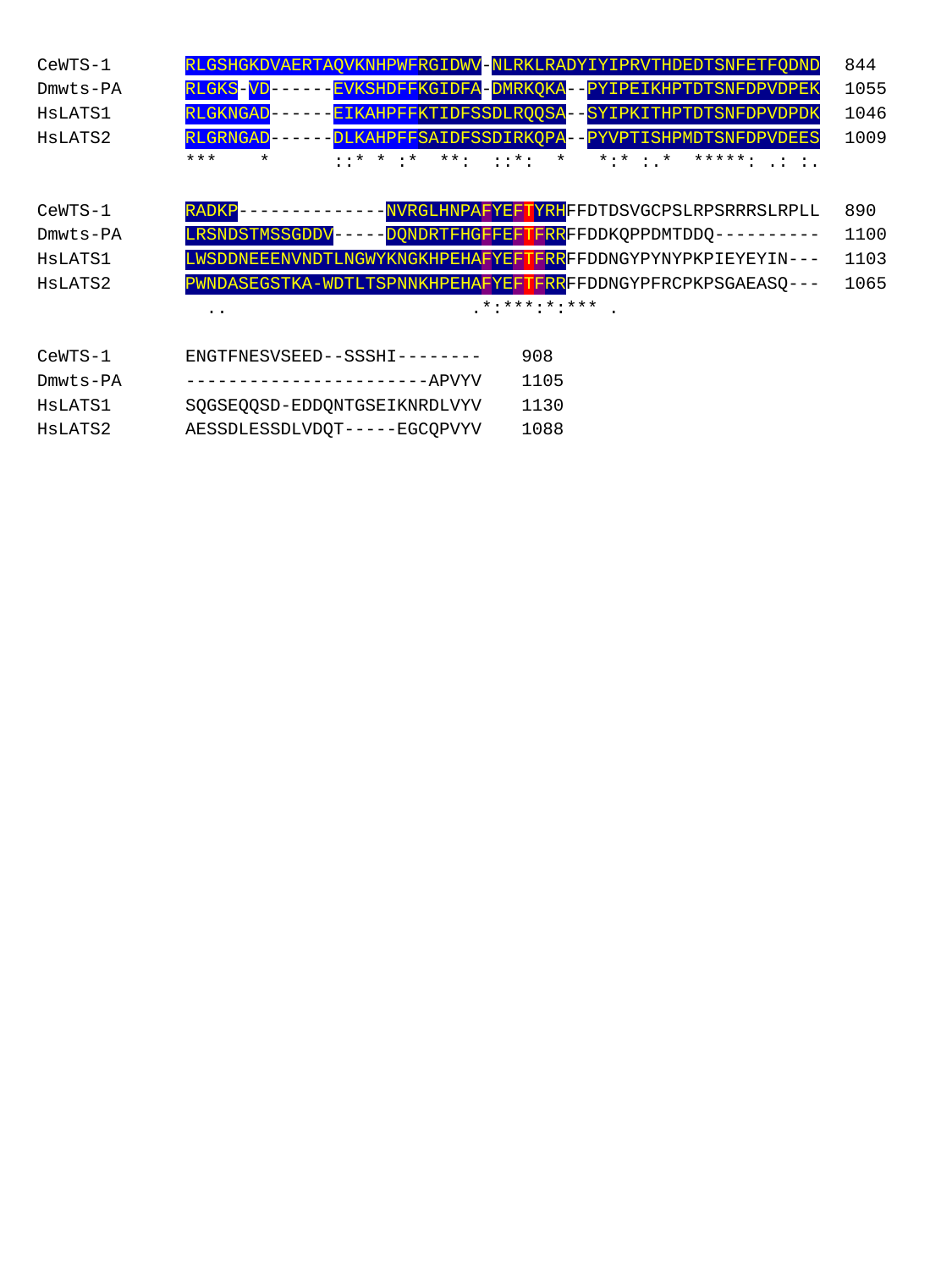

CeWTS-1 RLGSHGKDVAERTAQVKNHPWFRGIDWV-NLRKLRADYIYIPRVTHDEDTSNFETFQDND	844
Dmwts-PA RLGKS-VD------EVKSHDFFKGIDFA-DMRKQKA--PYIPEIKHPTDTSNFDPVDPEK	1055
HsLATS1 RLGKNGAD------EIKAHPFFKTIDFSSDLRQQSA--SYIPKITHPTDTSNFDPVDPDK	1046
HsLATS2 RLGRNGAD------DLKAHPFFSAIDFSSDIRKQPA--PYVPTISHPMDTSNFDPVDEES	1009
 *** * ::* * :* **: ::*: * *:* :.* *****: .: :.
CeWTS-1 RADKP--------------NVRGLHNPAFYEFTYRHFFDTDSVGCPSLRPSRRRSLRPLL	890
Dmwts-PA LRSNDSTMSSGDDV-----DQNDRTFHGFFEFTFRRFFDDKQPPDMTDDQ----------	1100
HsLATS1 LWSDDNEEENVNDTLNGWYKNGKHPEHAFYEFTFRRFFDDNGYPYNYPKPIEYEYIN---	1103
HsLATS2 PWNDASEGSTKA-WDTLTSPNNKHPEHAFYEFTFRRFFDDNGYPFRCPKPSGAEASQ---	1065
 .. .*:***:*:*** .
CeWTS-1 ENGTFNESVSEED--SSSHI--------	908
Dmwts-PA -----------------------APVYV	1105
HsLATS1 SQGSEQQSD-EDDQNTGSEIKNRDLVYV	1130
HsLATS2 AESSDLESSDLVDQT-----EGCQPVYV	1088

## Slide 6
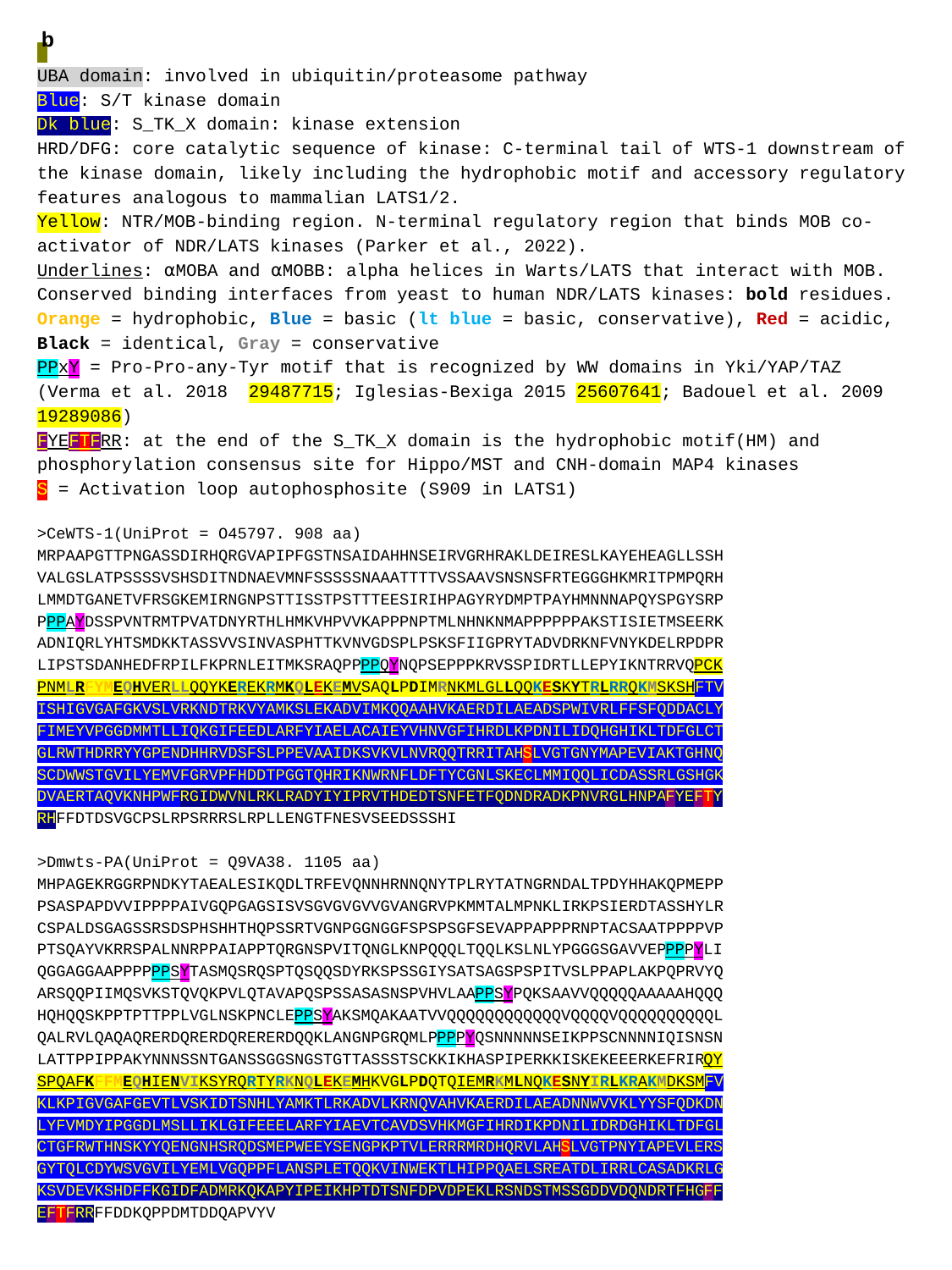

b
UBA domain: involved in ubiquitin/proteasome pathway
Blue: S/T kinase domain
Dk blue: S_TK_X domain: kinase extension
HRD/DFG: core catalytic sequence of kinase: C-terminal tail of WTS-1 downstream of the kinase domain, likely including the hydrophobic motif and accessory regulatory features analogous to mammalian LATS1/2.
Yellow: NTR/MOB-binding region. N-terminal regulatory region that binds MOB co-activator of NDR/LATS kinases (Parker et al., 2022).
Underlines: ⍺MOBA and ⍺MOBB: alpha helices in Warts/LATS that interact with MOB.
Conserved binding interfaces from yeast to human NDR/LATS kinases: bold residues. Orange = hydrophobic, Blue = basic (lt blue = basic, conservative), Red = acidic, Black = identical, Gray = conservative
PPxY = Pro-Pro-any-Tyr motif that is recognized by WW domains in Yki/YAP/TAZ (Verma et al. 2018 29487715; Iglesias-Bexiga 2015 25607641; Badouel et al. 2009 19289086)
FYEFTFRR: at the end of the S_TK_X domain is the hydrophobic motif(HM) and phosphorylation consensus site for Hippo/MST and CNH-domain MAP4 kinases
S = Activation loop autophosphosite (S909 in LATS1)
>CeWTS-1(UniProt = O45797. 908 aa)
MRPAAPGTTPNGASSDIRHQRGVAPIPFGSTNSAIDAHHNSEIRVGRHRAKLDEIRESLKAYEHEAGLLSSHVALGSLATPSSSSVSHSDITNDNAEVMNFSSSSSNAAATTTTVSSAAVSNSNSFRTEGGGHKMRITPMPQRHLMMDTGANETVFRSGKEMIRNGNPSTTISSTPSTTTEESIRIHPAGYRYDMPTPAYHMNNNAPQYSPGYSRPPPPAYDSSPVNTRMTPVATDNYRTHLHMKVHPVVKAPPPNPTMLNHNKNMAPPPPPPAKSTISIETMSEERKADNIQRLYHTSMDKKTASSVVSINVASPHTTKVNVGDSPLPSKSFIIGPRYTADVDRKNFVNYKDELRPDPRLIPSTSDANHEDFRPILFKPRNLEITMKSRAQPPPPQYNQPSEPPPKRVSSPIDRTLLEPYIKNTRRVQPCKPNMLRFYMEQHVERLLQQYKEREKRMKQLEKEMVSAQLPDIMRNKMLGLLQQKESKYTRLRRQKMSKSHFTVISHIGVGAFGKVSLVRKNDTRKVYAMKSLEKADVIMKQQAAHVKAERDILAEADSPWIVRLFFSFQDDACLYFIMEYVPGGDMMTLLIQKGIFEEDLARFYIAELACAIEYVHNVGFIHRDLKPDNILIDQHGHIKLTDFGLCTGLRWTHDRRYYGPENDHHRVDSFSLPPEVAAIDKSVKVLNVRQQTRRITAHSLVGTGNYMAPEVIAKTGHNQSCDWWSTGVILYEMVFGRVPFHDDTPGGTQHRIKNWRNFLDFTYCGNLSKECLMMIQQLICDASSRLGSHGKDVAERTAQVKNHPWFRGIDWVNLRKLRADYIYIPRVTHDEDTSNFETFQDNDRADKPNVRGLHNPAFYEFTYRHFFDTDSVGCPSLRPSRRRSLRPLLENGTFNESVSEEDSSSHI
>Dmwts-PA(UniProt = Q9VA38. 1105 aa)
MHPAGEKRGGRPNDKYTAEALESIKQDLTRFEVQNNHRNNQNYTPLRYTATNGRNDALTPDYHHAKQPMEPPPSASPAPDVVIPPPPAIVGQPGAGSISVSGVGVGVVGVANGRVPKMMTALMPNKLIRKPSIERDTASSHYLRCSPALDSGAGSSRSDSPHSHHTHQPSSRTVGNPGGNGGFSPSPSGFSEVAPPAPPPRNPTACSAATPPPPVPPTSQAYVKRRSPALNNRPPAIAPPTQRGNSPVITQNGLKNPQQQLTQQLKSLNLYPGGGSGAVVEPPPPYLIQGGAGGAAPPPPPPSYTASMQSRQSPTQSQQSDYRKSPSSGIYSATSAGSPSPITVSLPPAPLAKPQPRVYQARSQQPIIMQSVKSTQVQKPVLQTAVAPQSPSSASASNSPVHVLAAPPSYPQKSAAVVQQQQQAAAAAHQQQHQHQQSKPPTPTTPPLVGLNSKPNCLEPPSYAKSMQAKAATVVQQQQQQQQQQQQVQQQQVQQQQQQQQQQLQALRVLQAQAQRERDQRERDQRERERDQQKLANGNPGRQMLPPPPYQSNNNNNSEIKPPSCNNNNIQISNSNLATTPPIPPAKYNNNSSNTGANSSGGSNGSTGTTASSSTSCKKIKHASPIPERKKISKEKEEERKEFRIRQYSPQAFKFFMEQHIENVIKSYRQRTYRKNQLEKEMHKVGLPDQTQIEMRKMLNQKESNYIRLKRAKMDKSMFVKLKPIGVGAFGEVTLVSKIDTSNHLYAMKTLRKADVLKRNQVAHVKAERDILAEADNNWVVKLYYSFQDKDNLYFVMDYIPGGDLMSLLIKLGIFEEELARFYIAEVTCAVDSVHKMGFIHRDIKPDNILIDRDGHIKLTDFGLCTGFRWTHNSKYYQENGNHSRQDSMEPWEEYSENGPKPTVLERRRMRDHQRVLAHSLVGTPNYIAPEVLERSGYTQLCDYWSVGVILYEMLVGQPPFLANSPLETQQKVINWEKTLHIPPQAELSREATDLIRRLCASADKRLGKSVDEVKSHDFFKGIDFADMRKQKAPYIPEIKHPTDTSNFDPVDPEKLRSNDSTMSSGDDVDQNDRTFHGFFEFTFRRFFDDKQPPDMTDDQAPVYV

## Slide 7
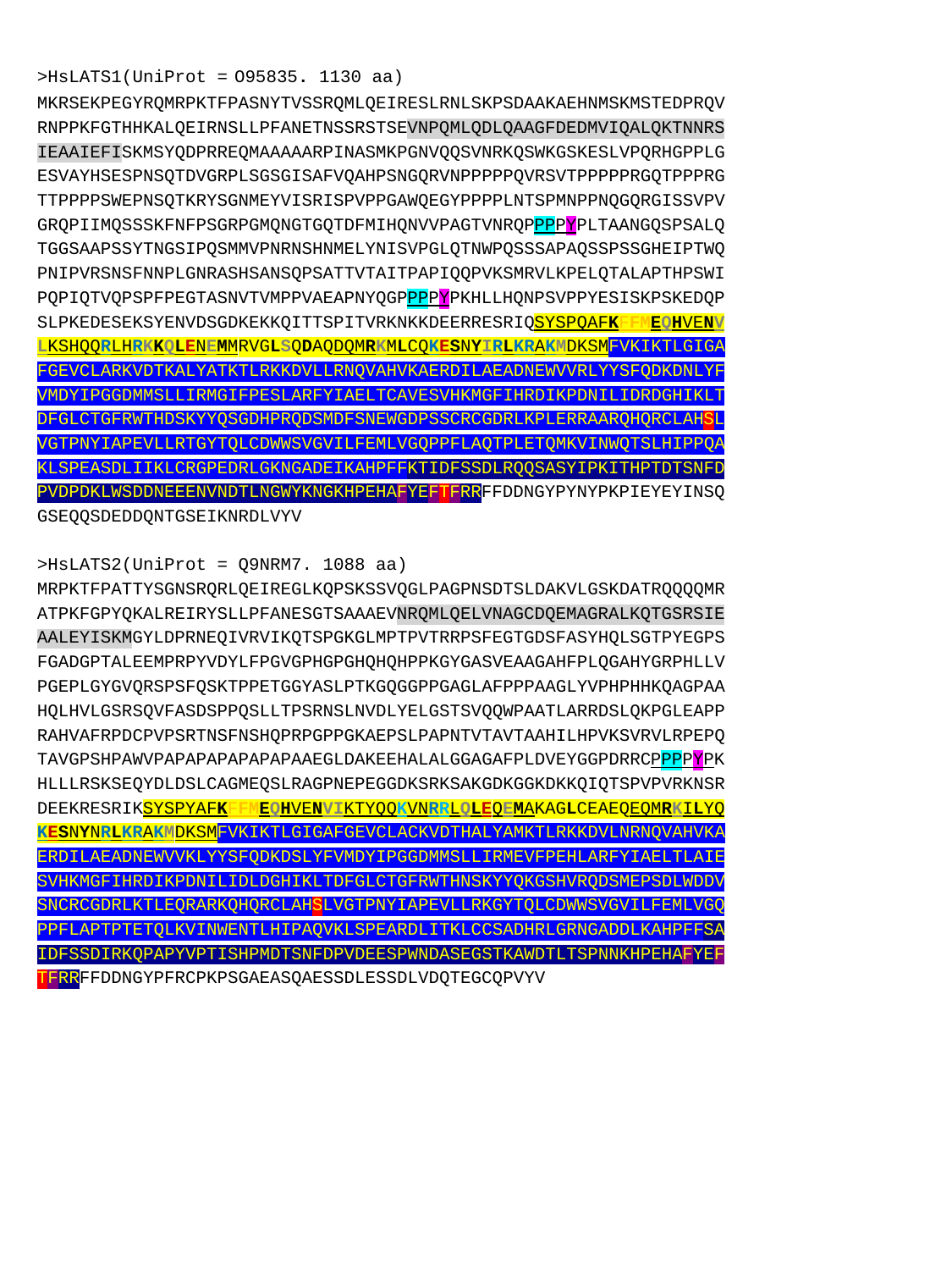

>HsLATS1(UniProt = O95835. 1130 aa)
MKRSEKPEGYRQMRPKTFPASNYTVSSRQMLQEIRESLRNLSKPSDAAKAEHNMSKMSTEDPRQVRNPPKFGTHHKALQEIRNSLLPFANETNSSRSTSEVNPQMLQDLQAAGFDEDMVIQALQKTNNRSIEAAIEFISKMSYQDPRREQMAAAAARPINASMKPGNVQQSVNRKQSWKGSKESLVPQRHGPPLGESVAYHSESPNSQTDVGRPLSGSGISAFVQAHPSNGQRVNPPPPPQVRSVTPPPPPRGQTPPPRGTTPPPPSWEPNSQTKRYSGNMEYVISRISPVPPGAWQEGYPPPPLNTSPMNPPNQGQRGISSVPVGRQPIIMQSSSKFNFPSGRPGMQNGTGQTDFMIHQNVVPAGTVNRQPPPPYPLTAANGQSPSALQTGGSAAPSSYTNGSIPQSMMVPNRNSHNMELYNISVPGLQTNWPQSSSAPAQSSPSSGHEIPTWQPNIPVRSNSFNNPLGNRASHSANSQPSATTVTAITPAPIQQPVKSMRVLKPELQTALAPTHPSWIPQPIQTVQPSPFPEGTASNVTVMPPVAEAPNYQGPPPPYPKHLLHQNPSVPPYESISKPSKEDQPSLPKEDESEKSYENVDSGDKEKKQITTSPITVRKNKKDEERRESRIQSYSPQAFKFFMEQHVENVLKSHQQRLHRKKQLENEMMRVGLSQDAQDQMRKMLCQKESNYIRLKRAKMDKSMFVKIKTLGIGAFGEVCLARKVDTKALYATKTLRKKDVLLRNQVAHVKAERDILAEADNEWVVRLYYSFQDKDNLYFVMDYIPGGDMMSLLIRMGIFPESLARFYIAELTCAVESVHKMGFIHRDIKPDNILIDRDGHIKLTDFGLCTGFRWTHDSKYYQSGDHPRQDSMDFSNEWGDPSSCRCGDRLKPLERRAARQHQRCLAHSLVGTPNYIAPEVLLRTGYTQLCDWWSVGVILFEMLVGQPPFLAQTPLETQMKVINWQTSLHIPPQAKLSPEASDLIIKLCRGPEDRLGKNGADEIKAHPFFKTIDFSSDLRQQSASYIPKITHPTDTSNFDPVDPDKLWSDDNEEENVNDTLNGWYKNGKHPEHAFYEFTFRRFFDDNGYPYNYPKPIEYEYINSQGSEQQSDEDDQNTGSEIKNRDLVYV
>HsLATS2(UniProt = Q9NRM7. 1088 aa)
MRPKTFPATTYSGNSRQRLQEIREGLKQPSKSSVQGLPAGPNSDTSLDAKVLGSKDATRQQQQMRATPKFGPYQKALREIRYSLLPFANESGTSAAAEVNRQMLQELVNAGCDQEMAGRALKQTGSRSIEAALEYISKMGYLDPRNEQIVRVIKQTSPGKGLMPTPVTRRPSFEGTGDSFASYHQLSGTPYEGPSFGADGPTALEEMPRPYVDYLFPGVGPHGPGHQHQHPPKGYGASVEAAGAHFPLQGAHYGRPHLLVPGEPLGYGVQRSPSFQSKTPPETGGYASLPTKGQGGPPGAGLAFPPPAAGLYVPHPHHKQAGPAAHQLHVLGSRSQVFASDSPPQSLLTPSRNSLNVDLYELGSTSVQQWPAATLARRDSLQKPGLEAPPRAHVAFRPDCPVPSRTNSFNSHQPRPGPPGKAEPSLPAPNTVTAVTAAHILHPVKSVRVLRPEPQTAVGPSHPAWVPAPAPAPAPAPAPAAEGLDAKEEHALALGGAGAFPLDVEYGGPDRRCPPPPYPKHLLLRSKSEQYDLDSLCAGMEQSLRAGPNEPEGGDKSRKSAKGDKGGKDKKQIQTSPVPVRKNSRDEEKRESRIKSYSPYAFKFFMEQHVENVIKTYQQKVNRRLQLEQEMAKAGLCEAEQEQMRKILYQKESNYNRLKRAKMDKSMFVKIKTLGIGAFGEVCLACKVDTHALYAMKTLRKKDVLNRNQVAHVKAERDILAEADNEWVVKLYYSFQDKDSLYFVMDYIPGGDMMSLLIRMEVFPEHLARFYIAELTLAIESVHKMGFIHRDIKPDNILIDLDGHIKLTDFGLCTGFRWTHNSKYYQKGSHVRQDSMEPSDLWDDVSNCRCGDRLKTLEQRARKQHQRCLAHSLVGTPNYIAPEVLLRKGYTQLCDWWSVGVILFEMLVGQPPFLAPTPTETQLKVINWENTLHIPAQVKLSPEARDLITKLCCSADHRLGRNGADDLKAHPFFSAIDFSSDIRKQPAPYVPTISHPMDTSNFDPVDEESPWNDASEGSTKAWDTLTSPNNKHPEHAFYEFTFRRFFDDNGYPFRCPKPSGAEASQAESSDLESSDLVDQTEGCQPVYV
