# Supplementary material for: Canonical and noncanonical Hippo signaling in C. elegans
Source: Genetics. 2026 Feb 26;233(1):iyag056. doi: 10.1093/genetics/iyag056 (PMC13147543; doi:10.1093/genetics/iyag056)
Supplement: iyag056_Supplementary_Data [file iyag056_supplementary_data.zip › Figure_S2_GENETICS-2025-308930.pptx]

## Slide 1
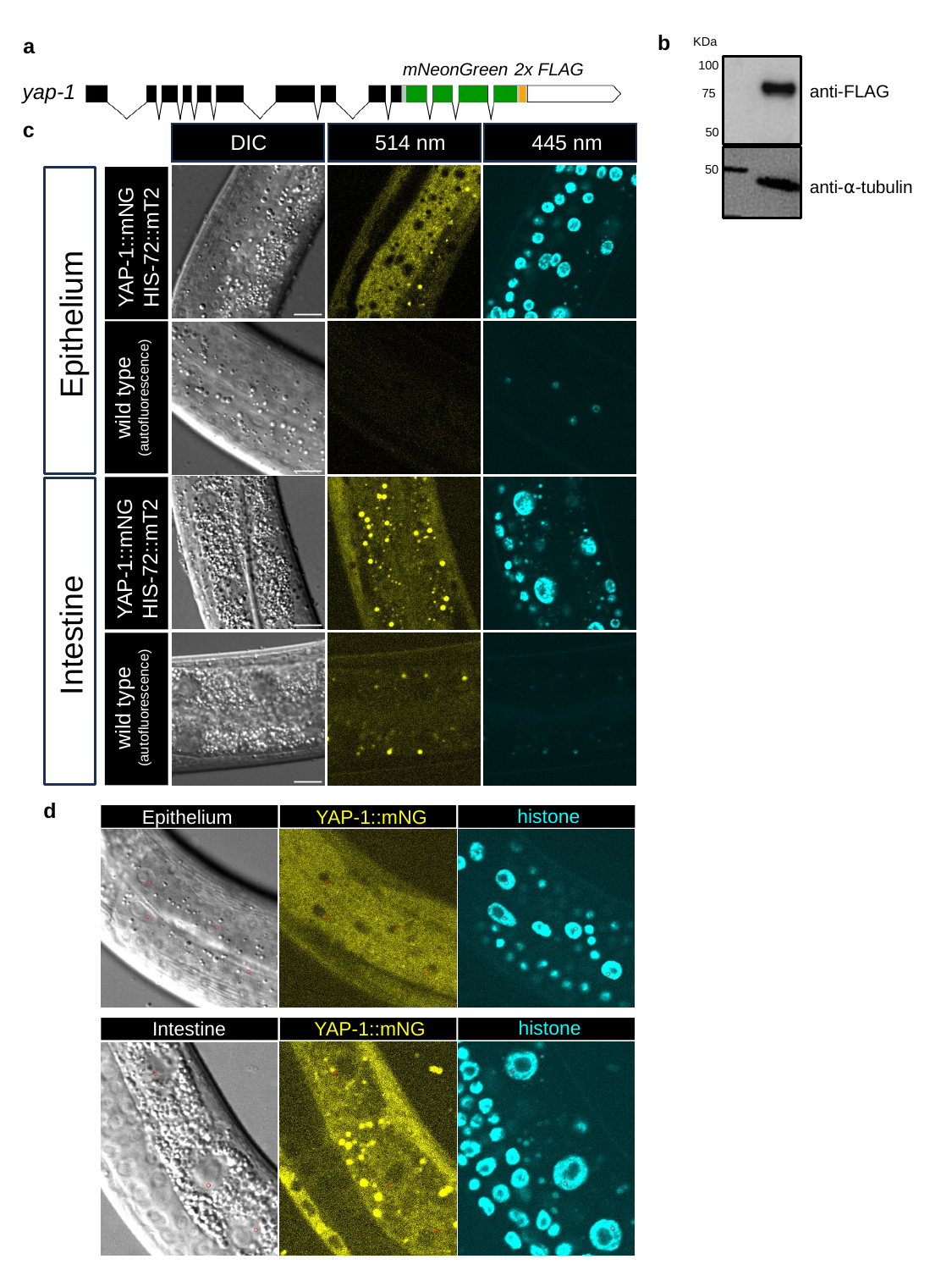

b
KDa
100
75
anti-FLAG
50
50
anti-⍺-tubulin
a
mNeonGreen
2x FLAG
yap-1
c
514 nm
445 nm
DIC
YAP-1::mNG
HIS-72::mT2
Epithelium
wild type
(autofluorescence)
YAP-1::mNG HIS-72::mT2
Intestine
wild type
(autofluorescence)
d
histone
Epithelium
YAP-1::mNG
histone
YAP-1::mNG
Intestine

## Slide 2
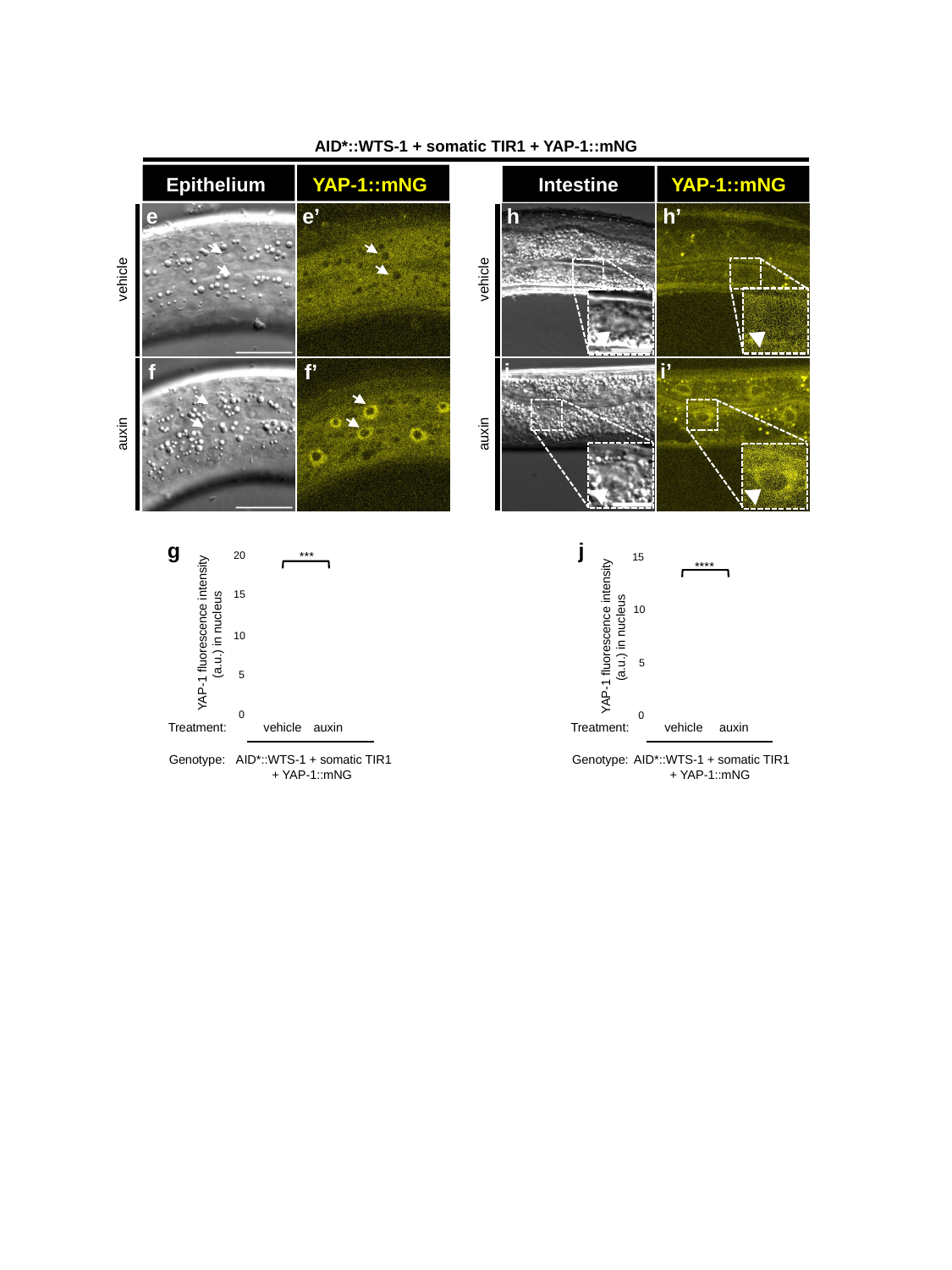

AID*::WTS-1 + somatic TIR1 + YAP-1::mNG
Epithelium
YAP-1::mNG
Intestine
YAP-1::mNG
e
e’
h
h’
vehicle
vehicle
i
i’
f
f’
auxin
auxin
g
j
20
***
15
****
15
10
YAP-1 fluorescence intensity (a.u.) in nucleus
YAP-1 fluorescence intensity (a.u.) in nucleus
10
5
5
0
0
Treatment:
vehicle
auxin
Treatment:
vehicle
auxin
Genotype:
AID*::WTS-1 + somatic TIR1 + YAP-1::mNG
Genotype:
AID*::WTS-1 + somatic TIR1 + YAP-1::mNG

## Slide 3
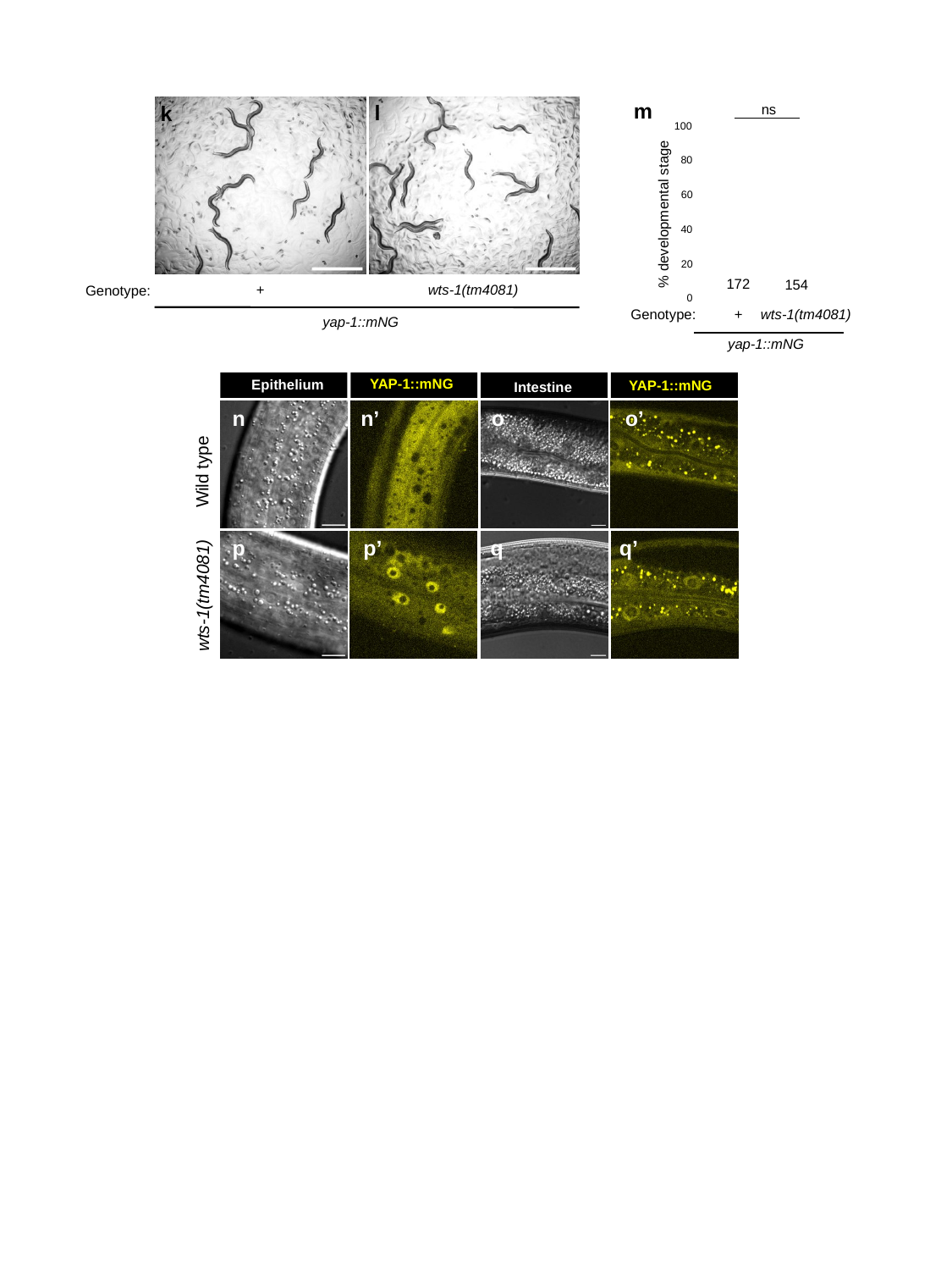

m
l
k
ns
100
80
60
% developmental stage
40
20
172
154
+
wts-1(tm4081)
Genotype:
 0
Genotype:
+
wts-1(tm4081)
yap-1::mNG
yap-1::mNG
YAP-1::mNG
Epithelium
YAP-1::mNG
Intestine
n
n’
o
o’
Wild type
p
p’
q
q’
wts-1(tm4081)
