# Supplementary material for: Canonical and noncanonical Hippo signaling in C. elegans
Source: Genetics. 2026 Feb 26;233(1):iyag056. doi: 10.1093/genetics/iyag056 (PMC13147543; doi:10.1093/genetics/iyag056)
Supplement: iyag056_Supplementary_Data [file iyag056_supplementary_data.zip › Figure_S3_GENETICS-2025-308930.pptx]

## Slide 1
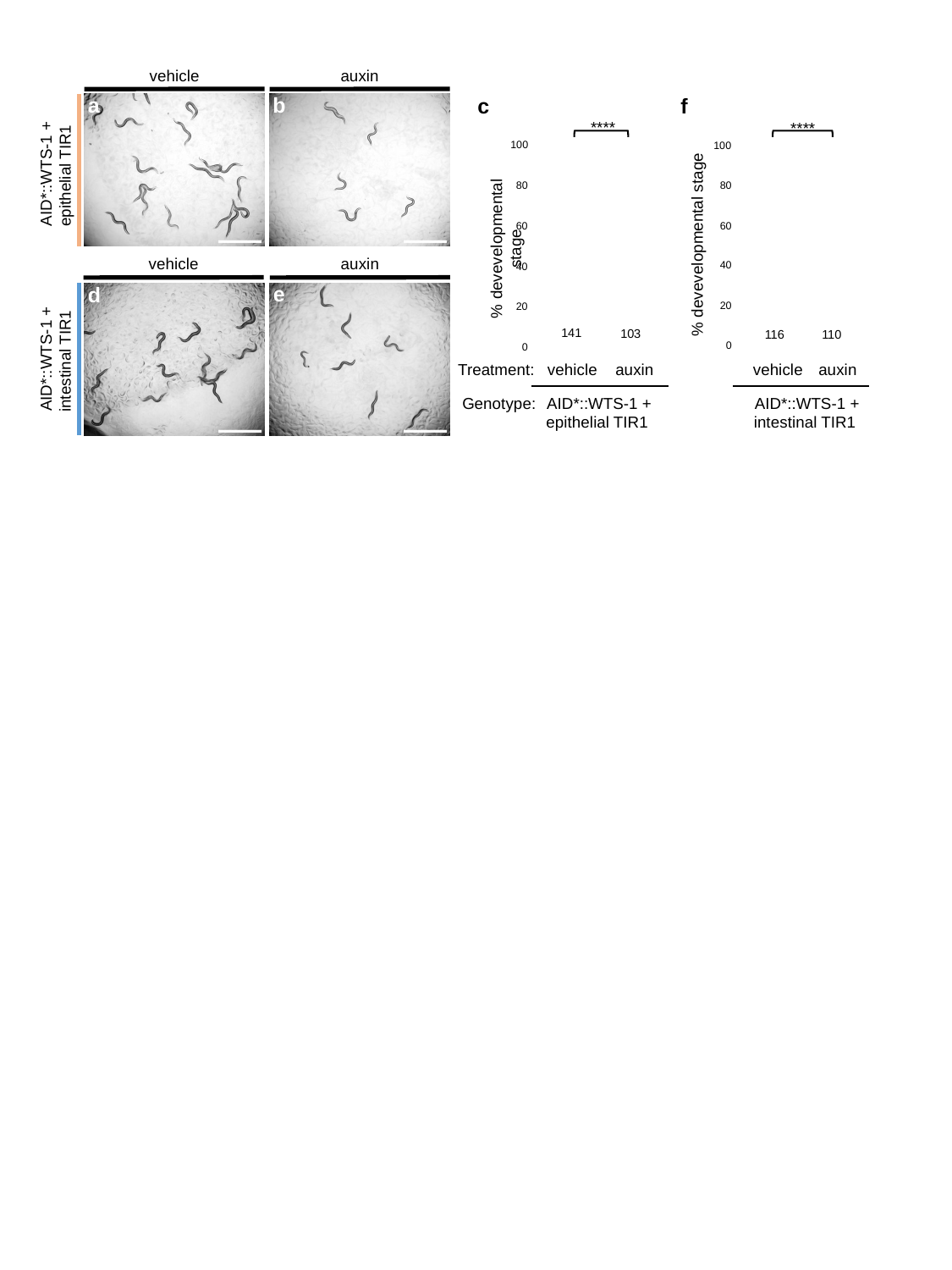

vehicle
auxin
a
b
c
f
****
****
100
100
AID*::WTS-1 + epithelial TIR1
80
80
60
60
% devevelopmental stage
% devevelopmental stage
vehicle
auxin
40
40
e
d
20
20
141
103
116
110
 0
AID*::WTS-1 + intestinal TIR1
 0
Treatment:
vehicle
auxin
vehicle
auxin
Genotype:
AID*::WTS-1 + epithelial TIR1
AID*::WTS-1 + intestinal TIR1
