# Supplementary material for: Canonical and noncanonical Hippo signaling in C. elegans
Source: Genetics. 2026 Feb 26;233(1):iyag056. doi: 10.1093/genetics/iyag056 (PMC13147543; doi:10.1093/genetics/iyag056)
Supplement: iyag056_Supplementary_Data [file iyag056_supplementary_data.zip › Figure_S4_GENETICS-2025-308930.pptx]

## Slide 1
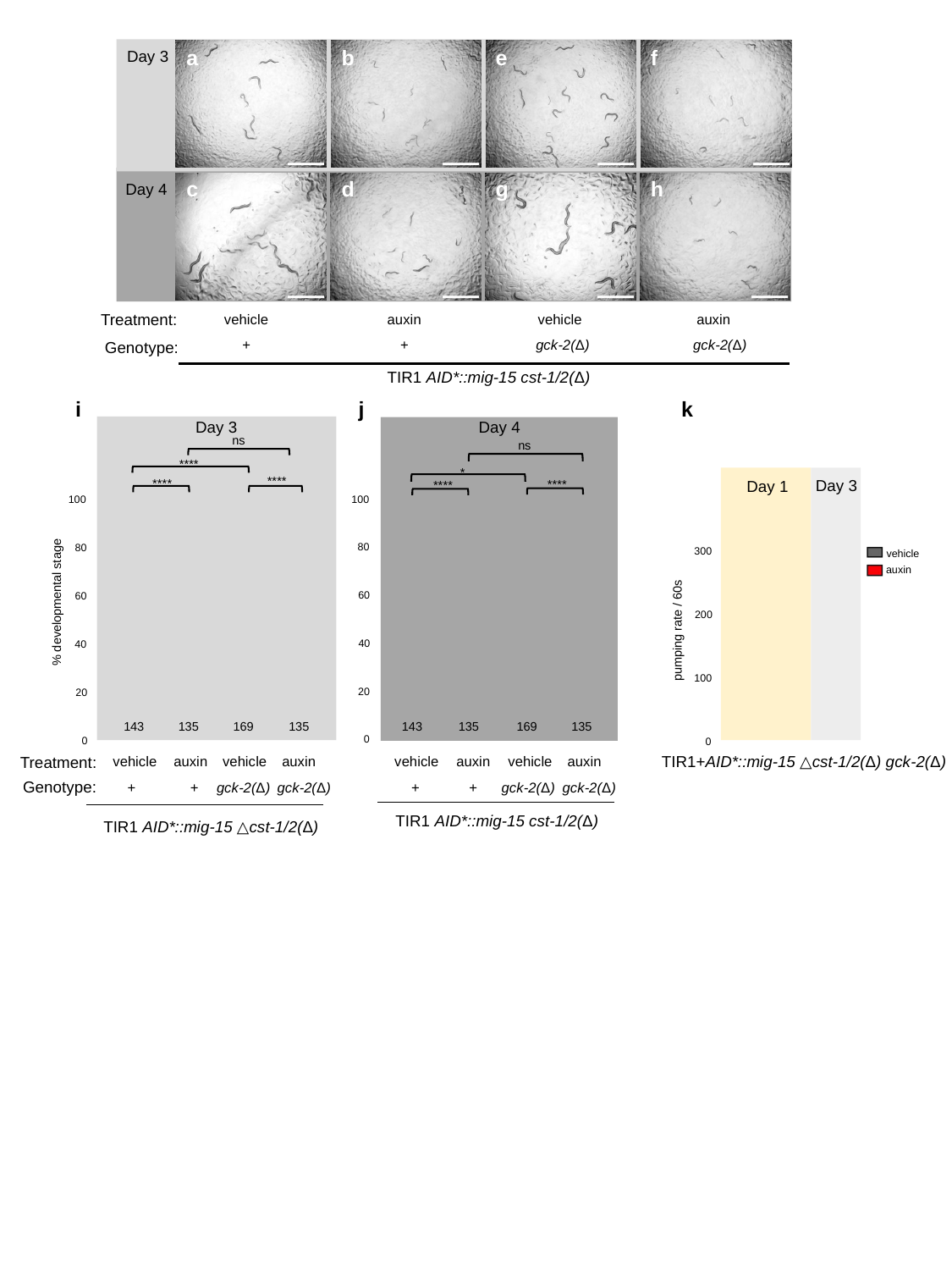

a
b
e
f
Day 3
c
d
g
h
Day 4
Treatment:
vehicle
auxin
vehicle
auxin
+
+
gck-2(Δ)
gck-2(Δ)
Genotype:
TIR1 AID*::mig-15 cst-1/2(Δ)
i
j
k
Day 3
Day 4
ns
ns
****
*
****
****
Day 3
Day 1
****
****
100
100
80
80
300
vehicle
auxin
60
60
% developmental stage
200
pumping rate / 60s
40
40
100
20
20
143
135
169
135
143
135
169
135
 0
 0
0
TIR1+AID*::mig-15 △cst-1/2(Δ) gck-2(Δ)
Treatment:
vehicle
auxin
vehicle
auxin
vehicle
auxin
vehicle
auxin
Genotype:
+
+
gck-2(Δ)
gck-2(Δ)
+
+
gck-2(Δ)
gck-2(Δ)
TIR1 AID*::mig-15 cst-1/2(Δ)
TIR1 AID*::mig-15 △cst-1/2(Δ)

## Slide 2
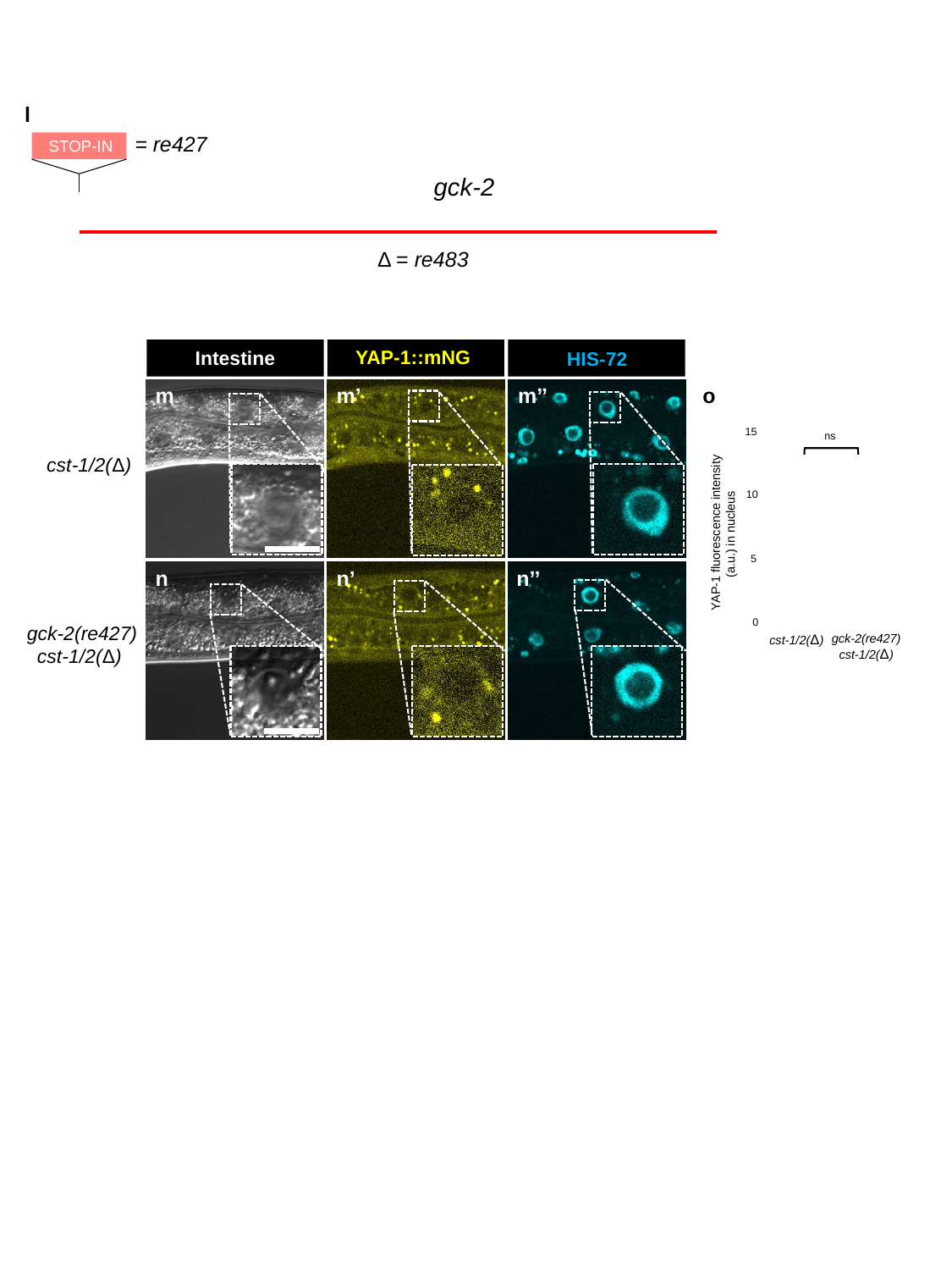

l
= re427
 STOP-IN
Δ = re483
gck-2
YAP-1::mNG
Intestine
HIS-72
m
m’
m’’
o
15
ns
cst-1/2(Δ)
10
YAP-1 fluorescence intensity (a.u.) in nucleus
5
n
n’
n’’
0
gck-2(re427) cst-1/2(Δ)
cst-1/2(Δ)
gck-2(re427) cst-1/2(Δ)
