# Supplementary material for: Canonical and noncanonical Hippo signaling in C. elegans
Source: Genetics. 2026 Feb 26;233(1):iyag056. doi: 10.1093/genetics/iyag056 (PMC13147543; doi:10.1093/genetics/iyag056)
Supplement: iyag056_Supplementary_Data [file iyag056_supplementary_data.zip › Figure_S5_GENETICS-2025-308930.pptx]

## Slide 1
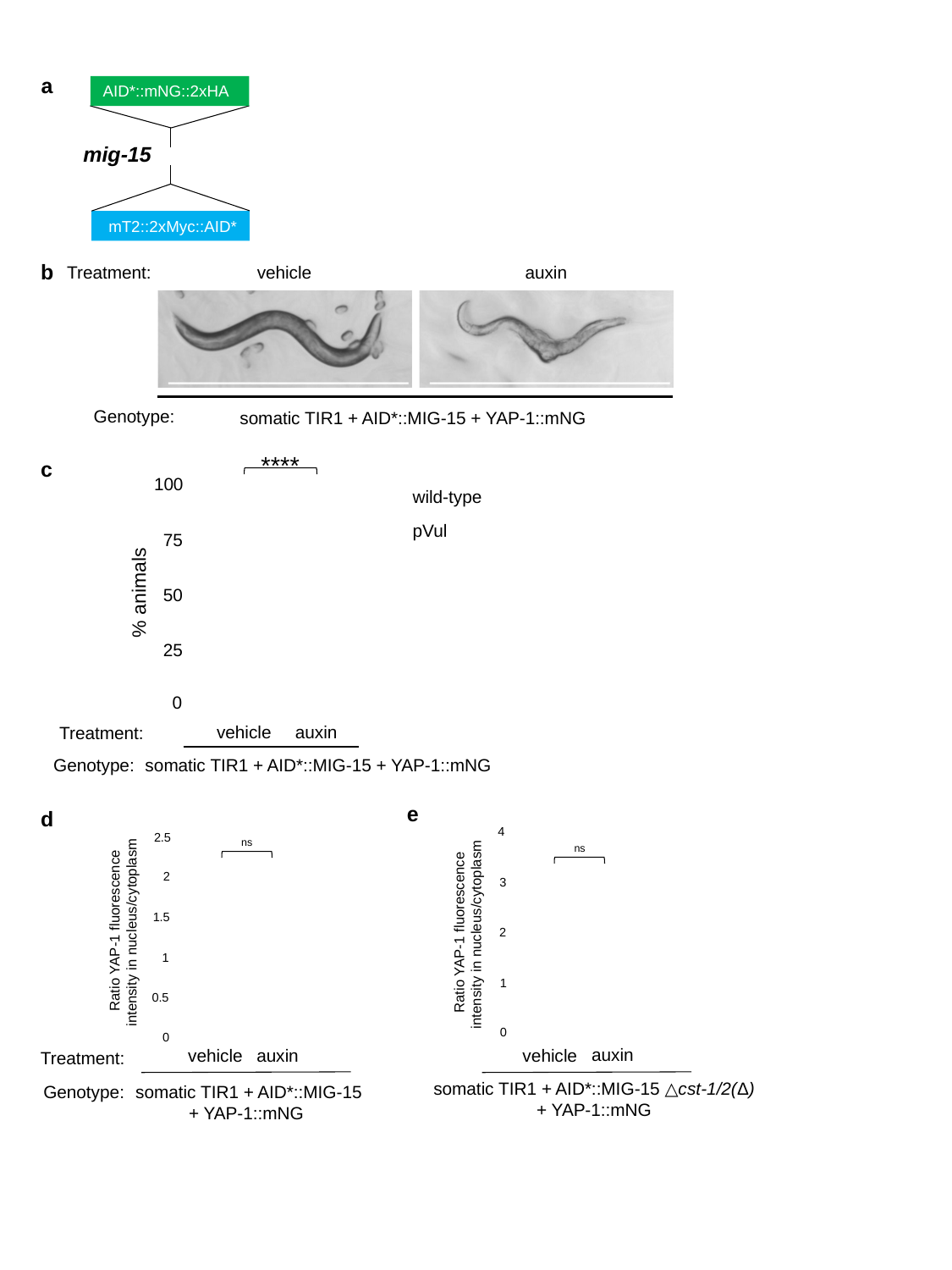

a
AID*::mNG::2xHA
mig-15
 mT2::2xMyc::AID*
b
auxin
vehicle
Treatment:
Genotype:
somatic TIR1 + AID*::MIG-15 + YAP-1::mNG
****
c
100
wild-type
pVul
75
% animals
50
25
125
153
153
125
0
vehicle
auxin
Treatment:
somatic TIR1 + AID*::MIG-15 + YAP-1::mNG
Genotype:
e
d
4
2.5
ns
ns
2
3
1.5
Ratio YAP-1 fluorescence intensity in nucleus/cytoplasm
Ratio YAP-1 fluorescence intensity in nucleus/cytoplasm
2
1
1
0.5
0
0
auxin
vehicle
vehicle
auxin
Treatment:
somatic TIR1 + AID*::MIG-15 △cst-1/2(Δ)
+ YAP-1::mNG
somatic TIR1 + AID*::MIG-15 + YAP-1::mNG
Genotype:
