# Supplementary material for: Canonical and noncanonical Hippo signaling in C. elegans
Source: Genetics. 2026 Feb 26;233(1):iyag056. doi: 10.1093/genetics/iyag056 (PMC13147543; doi:10.1093/genetics/iyag056)
Supplement: iyag056_Supplementary_Data [file iyag056_supplementary_data.zip › Supplemental_Figure_Legends_GENETICS-2025-308930.docx]

**Supplementary Figure Legends**

**Figure S1. Loss of WTS-1 confers YAP-1/YAP and EGL-44/TEAD-dependent developmental arrest. (a)** A schematic of the *wts-1* gene structure with deletion mutations and edits. The *wts-1(re436[STOP-IN])* deletes the start of the ATG initiator methionine codon and introduces stop codons in all three frames. (**b**) A schematic of the protein structure of WTS-1. Yellow shading indicates the NTR(“N-terminal region”)/MOB-binding domain, gray indicates the S/T kinase domain, and green indicates the kinase extension region. Red circles with a “P” indicate the sites of activating phosphorylation in other systems to shift the inhibitory loop and the activation phosphosite by Hippo/MST. PPxY motifs are WW-binding sites present in YAP and Yorkie. The region upstream of the yellow highlighting is generally proline-rich in all systems. **(c)** Quantification of developmental stage for *wts-1(re436[STOP-IN])* and *wts-1(ok753)* mutants on *ctrl-1(RNAi)* vs. *wts-1(RNAi*) on day 2, 23˚C. **(d)** Quantification of developmental stages of AID*::WTS-1 in germline-expressed TIR1 *ieSi68[sun-1p>TIR1::mRuby]* depleted with Auxin and *wts-1(RNAi)* on day 3. **(e-g)** No side effect of auxin treatment animals with somatic TIR1 but no AID* tag. **(h-k)** Growth delay stages of animals with depletion of AID*::WTS-1 + somatic TIR1 at Day 3 was reversed by *yap-1(RNAi)* or *egl-44(RNAi)*. **** = P<0.0001, *** = P<0.001, ** = P<0.01, ns = not significant (Fisher’s exact test). **(l)** Alignment of *C. elegans* WTS-1, *Drosophila* Warts, and Human LATS1 and LATS2 and annotation of conserved key domains and motifs. **(m)** Annotated sequences used for the alignment in **l**).

**Figure S2. Validation of tagged endogenous YAP-1. (a)** Schematic of the CRISPR-edited *yap-1* gene to create *yap-1(re269[yap-1::mNG::2xFLAG])*. Black = endogenous *yap-1*, green = sequences encoding mNeonGreen, orange = 2xFLAG. **(b)** Immunoblotting to detect endogenous YAP-1::mNG::2xFLAG at the expected 80.6 kDa. **(c)** YAP-1::mNG::2xFLAG and HIS-72::mT2 or untagged control signal detected in intestinal and epithelial planes at 514 nm or 445 nm. **(d)** The defined regions of interest (ROIs) for quantification are shown for both epithelial and intestinal confocal photomicrographs. Small red circles = ROIs. The animal genotype is *rrf-3(re390[STOP-IN])*; *his-72(erb77[his-72::linker::mTurquoise2])*; *yap-1(re269[yap-1::mNG::2xFLAG])* on luciferase RNAi. **(e-g)** Confocal and DIC photomicrographs and quantification of lateral epithelium; animals are oriented side-up. Animal genotype is *ieSi57[eft-3p::TIR1::mRuby::unc-54 3’UTR + Cbr-unc-119(+)]*; *wts-1(re419[mT2::2xMyc::AID*::wts-1])*; *yap-1(re269[yap-1::mNG::2xFLAG])*. **(g)** Vehicle vs. auxin treatment reveals nuclear translocation of YAP-1::mNG in epithelium as measured by arbitrary units (A.U.). **(h-j)** Confocal and DIC photomicrographs and quantification of intestinal midline; animals are oriented side-up. **(j)** Vehicle vs. auxin treatment reveals nuclear translocation of YAP-1::mNG in epithelium as measured by arbitrary units (A.U.). *yap-1* tagging interfered with *wts-*dependent L2 arrest, permitting animals to grow to age to be imaged. **(k-n)** Epifluorescent plate photomicrograph and quantification of animals of genotype *wts-1(+)*; *yap-1(re269[yap-1::mNG::2xFLAG])* **(k)** vs. *wts-1(tm4081)*; *yap-1(re269[yap-1::mNG::2xFLAG])* **(l)***.* **(m)** Quantification of developmental stages of homozygous *wts-1(tm4081)* compared to wild-type *wts-1* in *yap-1::mNG* background. We conclude the C-terminal tag of YAP-1 interferes with function as a transcription factor but still permits nuclear translocation as a reporter of WTS-1 activity. **(n-q’)** DIC and confocal photomicrographs of epithelium and intestine of tagged endogenous YAP-1 animals with and without deleted *wts-1*. **(n,n’)** *wts-1(*+*); yap-1(re269[yap-1::mNG::2xflag])* epithelium and **(o,o’)** intestine. **(p,p’)** *wts-1(tm4081)*; *yap-1(re269[yap-1::mNG::2xflag])* epithelium and **(q,q’)** intestine. We conclude the C-terminal tag of YAP-1 interferes with function as a transcription factor but still permits nuclear translocation as a reporter of WTS-1 activity. ns = not significant (Fisher’s exact test).

**Figure S3. Tissue-specific auxin depletion of WTS-1 without RNAi depletion. (a,b)** Bright field photomicrographs of animals with auxin-only AID*::WTS-1 depletion: no RNAi but epithelial-specific AID* depletion via epithelial-specific expression of TIR1. Genotype is *wts-1(re419[mT2::2xMyc::AID*::wts-1]); reSi2[col10p>TIR1::F2A::mTagBFP2::AID*::NLS::tbb-2 3'UTR]*. **(a)** Animals grown on vehicle *vs.* **(b)** auxin. **(c)** Quantification of **(a** vs. **b)**. **(d,e)** Bright field photomicrographs of animals with auxin-only AID*::WTS-1 depletion: no RNAi but intestinal-specific AID* depletion via intestinal-specific expression of TIR1. Genotype is *wts-1(re419[mT2::2xMyc::AID*::wts-1])*; *reSi12[ges1p>TIR1::F2A::mTagBFP2::AID*::NLS::tbb-2 3'UTR]*. **(d)** Animals grown on vehicle *vs.* **(e)** auxin. **(f)** Quantification of **d** vs. **e**. Scale bars = 1 mm. **** = P<0.0001 (Fisher’s exact test).

**Figure S4: Experiments for Fig. 5 but *gck-2* is also deleted*.* (a-h)** Bright-field photomicrographs of auxin-depleted AID*::MIG-15 *cst-1/2(*Δ*)* with and without *gck-2(re483)*, scored on day 3 (**a,b,e,f**) and day 4 (**c,d,g,h**) to illustrate degree of growth delay/arrest. Genotypes are *ieSi57[eft-3p>TIR1::mRuby::unc-54 3’UTR + Cbr-unc-119(+)]*; *unc-119(ed3)*; *mig-15(re264[AID*::mNG::2xHA::mig-15]) cst-1/2(reDf1[re484])* (**a-d**) *vs. ieSi57[eft-3p>TIR1::mRuby::unc-54 3’UTR + Cbr-unc-119(+)]*; *unc-119(ed3)*; *gck-2(re483)*; *mig-15(re264[AID*::mNG::2xHA::mig-15]) cst-1/2(reDf1[re484])* (**e-h**). Scale bar = 1 mm. **(i-j)** Quantification of stages of developmental of different genotypes in **a-h**, day 3 *vs.* day 4, respectively. The same animals shown in A,B,C,D were shown in Fig 5E,F,G,G to enable direct juxtaposition of animals with and without mutant *gck-2*. All were scored concurrently. **(k)** Pumping rate of animals with auxin-depleted AID*::mig-15 *cst-1/2(*Δ*) gck-2(re483)* scored on day 1 and day 3. **(l)** Schematic of *gck-2* will full-length deletion *gck-2(re483)* and STOP-IN insertion *gck-2(re427)* shown. **(m-n’’)** Confocal and DIC photomicrographs of intestinal YAP-1::mNG in *cst-1/2(*Δ*)* vs. *gck-2(re427) cst-1/2(*Δ*)* mutants, respectively. **(o)** Quantification of nuclear YAP-1::mNG in M’ vs N’, *cst-1/2(*Δ*)* with and without *gck-2(re427)*. N=10 animals for each group. **** = P<0.0001, *** = P<0.001, ** = P<0.01, ns = not significant. Scale bar of gene structure = 100 bp. Pop out intestinal scale bar = 10 μm.

**Figure S5. A second AID* tag of MIG-15.** **(a)** A schematic of the *mig-15* locus. The same tagging strategy used for the original MIG-15 tag with AID*::mNG::2xHA (Fakieh and Reiner 2025) as for mTurquoise2::2xMyc::AID*. Scale bar = 11 bp. **(b)** Auxin depletion of single mutant animals caused mild locomotion defects, slight delay in growth, and protruding vulva (pVul). Scale bar = 1 mm. **(c)** Quantification of the pVul defect. **(d**) Comparison of the ratios of intensity of nucleus to cytoplasm to nucleus in epithelium and **(e)** intestine, respectively, from **Figure 6 a-b’**, **d-e’**, and **h-i’**.
