# Supplementary material for: Canonical and noncanonical Hippo signaling in C. elegans
Source: Genetics. 2026 Feb 26;233(1):iyag056. doi: 10.1093/genetics/iyag056 (PMC13147543; doi:10.1093/genetics/iyag056)
Supplement: iyag056_Supplementary_Data [file iyag056_supplementary_data.zip › Supplemental_Tables_GENETICS-2025-308930.docx]

SUPPLEMENTARY TABLES

**Table S1: Strains**

| **Strain** | **Genotype** |
| --- | --- |
| CA1200 | *ieSi57[eft-3p>TIR1::mRuby + Cbr-unc-119(+)]* II; *unc-119(ed3)* III |
| DV3709 | *ieSi57[eft-3p>TIR1::mRuby + Cbr-unc-119(+)]* II; *unc-119(ed3)* III; *mig-15(re264[AID*::mNG::2xHA::mig-15])* X |
| DV3948 | *ieSi57[eft-3p>TIR1::mRuby + Cbr-unc-119(+)]* II; *unc-119(ed3)* III; *mig-15(re264[AID*::mNG::2xHA::mig-15]) cst-1/2(reDf1[re484])* X |
| DV3969 | *ieSi57[eft-3p>TIR1::mRuby + Cbr-unc-119(+)]* II; *unc-119(ed3)* III; *gck-2(re483)* V; *mig-15(re264[AID*::mNG::2xHA::mig-15]) cst-1/2(reDf1[re484])* X |
| DV3611 | *yap-1(re269[yap-1::mNG::2xflag])* X |
| DV4205 | *ieSi57[eft-3p>TIR1::mRuby + Cbr-unc-119(+)]* II; *unc-119(ed3)* III; *yap-1(re269[yap-1::mNG::2xflag])* X |
| DV4130 | *rrf-3(re390[STOP-IN*])* II; *his-72 (erb77[his-72::linker::mTurquoise2])* III, *yap-1(re269[yap-1::mNG::2xflag]* X |
| DV4186 | *his-72(erb77[his-72::linker::mTurquoise2])* III, *yap-1(re269[yap-1::mNG::2xflag] cst-1/2(reDf4[re452])* X |
| DV4217 | *wts-1(tm4081)*/*tmC18[dpy-5(tmls1236[myo-2p>mCherry])]* I |
| DV4214 | *wts-1(re419[mTurqoise2::2xMyc::AID*::wts-1])* I; *ieSi57[eft-3p>TIR1::mRuby + Cbr-unc-119(+)]* II; *yap-1(re269[yap-1::mNG::2xflag]* X |
| DV4288 | *his-72(erb77[his-72::linker::mTurquoise2])* III; *gck-2(re427[STOP-IN*])* V; *yap-1(re269[yap-1::mNG::2xflag]) cst-1/2(reDf4[re452])* X |
| DV4290 | *wts-1(re419[mTurqoise2::2xMyc::AID*::wts-1])* I; *ieSi57[eft-3p>TIR1::mRuby + Cbr-unc-119(+)]* II; *unc-119(ed3)* III |
| DV4292 | *wts-1(ok753)*/*tmC18[dpy-5(tmls1236[myo-2p>mCherry])]* I |
| DV4278 | *wts-1(tm4081)* I; *yap-1(re269[yap-1::mNG::2xflag])* X |
| DV4297 | *wts-1(re436[STOP-IN*])*/*tmC18[dpy-5(tmls1200[myo-2p>Venus])* I |
| DV4296 | *ieSi57[eft-3p>TIR1::mRuby + Cbr-unc-119(+)]* II*; unc-119(ed3)* III; *mig-15(re435[mTurquoise2::2xmyc::AID*::mig-15) yap-1(re269[yap-1::mNG::2xflag])* X |
| DV4337 | *wts-1(re419[mTurqoise2::2xmyc::AID*::wts-1])* I; *reSi2[col-10p>TIR1::F2A::mTaqBFP2::AID::NLS]* II |
| DV4338 | *wts-1(re419[mTurqoise2::2xmyc::AID*::wts-1])* I; *reSi12[ges-1p>TIR1::F2A::mTaqBFP2::AID*::NLS]* II |
| DV4339 | *wts-1(re419[mTurqoise2::2xmyc::AID*::wts-1])* I; *reSi2[col-10p>TIR1::F2A::mTaqBFP2::AID*::]* II; *yap-1(re269[yap-1::mNG::2xflag])* X |
| DV4340 | *wts-1(re419[mTurqoise2::2xmyc::AID*::wts-1])* I; *reSi12[ges-1p>TIR1::F2A::mTaqBFP2::AID*::NLS]* II; *yap-1(re269[yap-1::mNG::2xflag])* X |
| DV4341 | *wts-1(re419[mTurqoise2::2xmyc::AID*::wts-1])* I; *ieSi68[sun-1p>TIR1::mRuby + Cbr-unc-119(+)]* II |
| DV4384 | *ieSi57[eft-3p>TIR1::mRuby + Cbr-unc-119(+)]* II*; unc-119(ed3)* III; *mig-15(re435[mTurquoise2::2xmyc::AID*::mig-15) yap-1(re269[yap-1::mNG::2xflag]) cst-1/2(re479)* X |

**Table S2: Oligonucleotides**

| **Name** | **Oligonucleotide sequence** | **Use** |
| --- | --- | --- |
| LH12 | GATATTGCCGAGCACACATGG | genotyping triplex *rrf-3(re390)* |
| DJR881-RV1 | GCTTATCACTTAGTCACCTCTGCTC | genotyping triplex *rrf-3(re390)* |
| LH13 | GATGTGAAACTTGATGCGAACTCG | genotyping triplex *rrf-3(re390)* |
| LH11 repair *rrf-3* | AATTTCAGTCTAAAGTTGACGAGGTAAACTGGATCAGGGAAGTTTGTCCAGAGCAGAGGTGACTAAGTGATAAGCTAGCAGGTGGGGATGGTGCCACGAGCTGCGTACGAAGATAAA | repair template for *rrf-3(re390)* |
| LH60 | CTTACTTTCTATTCAAGGTTTTATTTCAAACGTAGGACACC | genotyping triplex *wts-1(tm4081)* |
| LH61 | TGCTGTAGAAGTCGTTCAACATGTTGTTCC | genotyping triplex *wts-1(tm4081)* |
| LH62 | TGTCTTCGCAGACACCCATAATCAGC | genotyping *triplex wts-1(tm4081)* |
| LH200 | AAAGCGGATGTCATAATGAAACAACAGGTAGG | genotyping triplex *wts-1(ok753)* |
| LH201 | GTGAAAGCCGAACGCGATATTCTGG | genotyping triplex *wts-1(ok753)* |
| LH202 | AGCTGCAACTTCAGGAGGAAGC | genotyping triplex *wts-1(ok753)* |
| LH131 | CGGGAAGCAAAGAGACCACATAACG | genotyping triplex *wts-1(re436[STOP IN])* and *wts-1(re419[mTurqoise2::2xmyc::AID*::wts-1])* |
| LH133 | AACCAAAACCTACTGAATTGTGATGGGC | genotyping triplex *wts-1(re436[STOP IN])* and *wts-1(re419[mTurqoise2::2xmyc::AID*::wts-1])* |
| DJR881-RV1 | GCTTATCACTTAGTCACCTCTGCTC | genotyping triplex *wts-1(re436[STOP IN])* |
| LH134 repair wts-1 stop-in | GTGTTCGGCAGGAGGCCCTCCTTTGCATTGTATTCCCGGGAAGTTTGTCCAGAGCAGAGGTGACTAAGTGATAAGCTAGCTGCGACCTGCTGCCCCTGGTACTACTCCAAAT | repair template for *wts-1(re436[STOP IN])* |
| LH132 RV1_wts-1-AID | CGGTAAAGAGTTCTTCTCCTTTGGAGACC | genotyping triplex *wts-1(re419[mTurqoise2::2xmyc::AID*::wts-1])* |
| LH100 FW_repair mTurq2-wts-1 | GGAGGCCCTCCTTTGCATTGTATTCCCCTGGAATGGTCTCTAAGGGAGAAGAACTCTTTACC | primer for amplifying fragment #1 repair template for *wts-1(re419[mTurqoise2::2xmyc::AID*::wts-1])* |
| LH101 RV_repair mTurq2-wts-1 | ATCAGAAGATGCACCATTTGGAGTAGTACCTGGAGCTGCAGGTCGCATacCtGCgCCgCTTGCTCCtGATCCaGCaCCCTTCACG | primer for amplifying fragment #2 repair template for *wts-1(re419[mTurqoise2::2xmyc::AID*::wts-1])* |
| CB003 Deletion Repair Template | CAAAACAAAGTGTTGAGATAGTGGATGAAAGTTTGCAGTGGCAATTCACGATTAAAGTCAAATACTAAAA | repair template for *cst-1/2(reDf1), cst-1/2(reDf4) and cst-1/2(re479)* |
| CB004 CST FW | GGCATCCGTTTCGACAGTATAG | genotyping triplex *cst-1/2(reDf1),* c*st-1/2(reDf4) and cst-1/2(re479)* |
| CB005 CST RV-1 | GCCACTGCAAACTTTCATCC | genotyping triplex *cst-1/2(reDf1),* c*st-1/2(reDf4) and cst-1/2(re479)* |
| CB006 CST RV-2 | TATGTGTCGTGGGAAAGTGC | genotyping triplex *cst-1/2(reDf1),* c*st-1/2(reDf4) and cst-1/2(re479)* |
| RF21 | TTAGCAACGATAACAATCGAGGAGTGGGATCAATGGTGCACCATTATCAAGGAGCATCGGGAGCCTCAG | primer for amplifying fragment #1 repair template for YAP-1 tag |
| RF22 | AGAGAAAGAGAGTGGTTATTCTGCTGATTAGACATTTACGAGGCTCCACGCTTGTCG | primer for amplifying fragment #2 repair template for YAP-1 tag |
| RF26 FW | CACACGAATTCGACCAGTATCTACA | genotyping *yap-1(re269[yap-1::mNG::2xflag])* |
| RF27 RV | CATCGACCATAGCGGCTTG | genotyping *yap-1(re269[yap-1::mNG::2xflag])* |
| RF28 RV | GTATTGGAAACGGGGTTGAGC | genotyping *yap-1(re269[yap-1::mNG::2xflag])* |
| LH004v2 GCK-2 Repair Template | GTGATTCACTGGCAGCTGTCAAAGTAGTCAAACTCGATCGCAACCAATGTCACCCCGAAACTCTGGCGATAC | repair template for *gck-2(re483)* |
| LH140 GCK-2 STOP-IN Repair Template | TGATTCACTGGCAGCTGTCAAAGTAGTCAAACTCGGGGAAGTTTGTCCAGAGCAGAGGTGACTAAGTGATAAGCTAGCAGGCGGGCGACAATTTTGCAGTAATCCAGCAGGAGATT | repair template for *gck-2(re427[STOP IN)* |
| LH80 GCK-2 FW | GGGTAATGAGTGCCGATGTAATCAAACG | genotyping triplex *gck-2(re483)* |
| LH81 GCK-2 RV-1 | GAAGGCGATTTGAAGTTCCGAAAGTGG | genotyping triplex *gck-2(re483)* |
| LH83 GCK-2 RV-2 | AGTTTCGGGGTGACATTGGTTGC | genotyping triplex *gck-2(re483)* |
| LH141 | GGTAATGAGTGCCGATGTAATCAAACG | genotyping triplex *gck-2(re427[STOP IN)* |
| DJR881 stop-in RV1 | GCTTATCACTTAGTCACCTCTGCTC | genotyping triplex *gck-2(re427[STOP IN)* |
| LH143 | GCCGCAGTACTCCATAACAATCC | genotyping triplex *gck-2(re427[STOP IN)* |
| LH148_FW primer of AID-mig-15 repair template | TTGCTCCAAGCCGCTCACAGCACCCAAAACCATGTCAATGGTCTCCAAAGGAGAAGAACTC | primer for amplifying fragment #1 repair template for *mig-15(re435[mTurquoise2::2xmyc::AID*::mig-15])* |
| LH149_RV primer of AID-mig-15 repair template | AGAATTCAAATCAATCTCGTCGAGTCCTGATGACGACATACCTGCGCCGCTTGCTCCTGATCCAGCACCCTTCACG | primer for amplifying fragment #2 repair template for *mig-15(re435[mTurquoise2::2xmyc::AID*::mig-15])* |
| RF15_FW_AID-MIG-15 | CATTGTGTGGTTTAAGTGTCGGC | genotyping triplex *mig-15(re435)* |
| LH151_RV1_AID-MIG-15 | GTGCAAATGAATTTAAGGGTGAGTTTTCC | genotyping triplex *mig-15(re435)* |
| LH152_RV2_AID-MIG-15 | CGTGTTGTTTGGTGTAGTTTCGC | genotyping triplex *mig-15(re435)* |
| *dpy-10(cn64*gf*)* ssODN repair template | CACTTGAACTTCAATACGGCAAGATGAGAATGACTGGAAACCGTACCGCATGCGGTGCCTATGGTAGCGGAGCTTCACATGGCTTCAGACCAACAGCCTAT | ssODN repair template for *dpy-10(cn64*gf*)* |

**Table S3: CRISPR guide RNAs**

| **RNA** | **Sequence** | **Use** |
| --- | --- | --- |
| LH10 rrf-3 crRNA-1 | CGAGGUAAACUGGAUCAAGGGUUUUAGAGCUAUGCUGUUUUG | crRNA for *rrf-3(re390)* |
| LH130 crRNA rev wts-1 | GGCAGCAGGUCGCAUUCCAGGUUUUAGAGCUAUGCUGUUUUG | crRNA for *wts-1(re436)* and *wts-1(re419)* |
| CB001 cst-2 crRNA | UAGUGGAUGAAAGUUUGGACGUUUUAGAGCUAUGCUGUUUUG | crRNA#1 for *cst-1/2(reDf1), cst-1/2(reDf4)* and *cst-1/2(re479)* |
| CB002 cst-1 crRNA | AUCGUGAAUUGCCACUGUUGGUUUUAGAGCUAUGCUGUUUUG | crRNA#2 for *cst-1/2(reDf1), cst-1/2(reDf4) and cst-1/2(re479)* |
| RF20 | CAATCGAGGAGTGGGATCAAGUUUUAGAGCUAUGCUGUUUUG | crRNA for C term tag *yap-1(re269[yap-1::mNG::2xflag])* |
| LH005 GCK-2 crRNA-1 | CAAAGUAGUCAAACUCGAGGGUUUUAGAGCUAUGCUGUUUUG | crRNA for *gck-2(re483) and gck-2(re427)* |
| LH006 GCK-2 crRNA-2 | GUGACAUUGGUUGCGAUCGGGUUUUAGAGCUAUGCUGUUUUG | crRNA for *gck-2(re483)* |
| crRNA guide for mig-15 5' end | GTCGAGTCCTGATGACGACAGUUUUAGAGCUAUGCUGUUUUG | crRNA for tag *mig-15(re435[mTurquoise2::2xmyc::AID*::mig-15])* |
| dpy-10 crRNA | GCUACCAUAGGCACCACGAGGUUUUAGAGCUAUGCUGUUUUG | crRNA for *dpy-10* |
| tracrRNA | AACAGCAUAGCAAGUUAAAAUAAGGCUAGUCCGUUAUCAACUUGAAAAAGUGGCACCGAGUCGGUGCUUUUUUU | universal |

**Table S4: Plasmids**

| **Plasmid** | **Use** | **Note** |
| --- | --- | --- |
| pNR pBS mNG::2xFLAG | tag *yap-1(re269[yap-1::mNG::2xflag])* | sequence verified |
| pYW2 pBS mTurq2::2xMYC::AID* | tag *wts-1(re419[mTurqoise2::2xmyc::AID*::wts-1])* and *mig-15(re435[mTurquoise2::2xmyc::AID*::mig-15])* | sequence verified |
| pREW2 | *luciferase*(*RNAi*) | sequence verified |
| I-5K13 | *wts-1(RNAi)* (T20F10.1) | sequence verified |
| X-5M19 | *yap-1(RNAi)* RNAi (F13E6.4) | sequence verified |
| X-11E16 | *egl-44(RNAi)* (F28B12.2a) | sequence verified |
| I-1K04 | *pop-1(RNAi)* (W10C8.2) | sequence verified |
